# Two-Photon Polymerization: Functionalized Microstructures, Micro-Resonators, and Bio-Scaffolds

**DOI:** 10.3390/polym13121994

**Published:** 2021-06-18

**Authors:** Adriano J. G. Otuka, Nathália B. Tomazio, Kelly T. Paula, Cleber R. Mendonça

**Affiliations:** 1 Photonics Group, São Carlos Institute of Physics, University of São Paulo, São Carlos 13566-590, SP, Brazil; nathalia.tomazio@alumni.usp.br (N.B.T.); kelly_tasso@hotmail.com (K.T.P.); 2 Device Research Laboratory, “Gleb Wataghin” Institute of Physics, University of Campinas, Campinas 13083-859, SP, Brazil

**Keywords:** direct laser writing, ultrashort laser pulses, two-photon polymerization, functional microdevices, whispering gallery mode microresonators, scaffolds for biological applications

## Abstract

The direct laser writing technique based on two-photon polymerization (TPP) has evolved considerably over the past two decades. Its remarkable characteristics, such as 3D capability, sub-diffraction resolution, material flexibility, and gentle processing conditions, have made it suitable for several applications in photonics and biosciences. In this review, we present an overview of the progress of TPP towards the fabrication of functionalized microstructures, whispering gallery mode (WGM) microresonators, and microenvironments for culturing microorganisms. We also describe the key physical-chemical fundamentals underlying the technique, the typical experimental setups, and the different materials employed for TPP.

## 1. Introduction

The processing of materials by ultrashort lasers, which allows high precision-patterning of 2D and 3D micro/nanostructures, has become a cornerstone technology for a wide range of areas related to academic research and engineering [1]. An ultrashort laser-based fabrication technique that has been widely used is direct laser writing via two-photon polymerization (TPP) [2,3]. As opposed to the most conventional micro/nanofabrication techniques, TPP is a maskless patterning tool and does not require harsh processing conditions nor a cleanroom facility [4]. Furthermore, this technique is not limited to the processing materials in the powder form, such as selective laser sintering (SLS) [5,6,7,8], another laser fabrication method that allows sculpting macroscopic structures with micrometric resolution. TPP leverages the nonlinear nature of two-photon absorption to fabricate 3D polymeric microstructures with arbitrary geometries and sub-diffraction features [9]. Moreover, a large variety of materials can be employed as photoresist, or be incorporated into it, which allows customizing the microstructures’ physical, chemical or biological properties to meet specific applications [10,11]. For example, improvements in the spatial resolution and the amount of dry-related shrinkage undergone by the microstructures can be accomplished by varying the photoresist components proportion [1,12,13,14].

The TPP technique has found applications in numerous fields, including photonics [15,16,17,18,19], biosciences [20,21,22], and micromechanical systems [23,24,25]. Precise photonic building blocks, such as optical microresonators [26], photonic crystals [27], and waveguides [28], as well as photonic wire bondings [29] and free-form coupling elements [16], have been successfully fabricated by TPP. Active photonic structures have also been fabricated [30,31]. In the field of biological sciences, TPP has mainly been used to create microscaffolds suitable for culturing cells, tissues, and microorganisms [21,32,33], and microneedles [22] and porous microstructures [20] for drug delivery. TPP has also been a remarkable tool for fabricating optically driven micromachines that allows grabbing or probing nanoscale objects [34]. Microstructures containing movable parts, such as nanotweezers [23], microgears [24], and micropump setups [35], have been demonstrated.

This paper summarizes the advances of TPP towards three of its major applications: functionalized microstructures, whispering gallery mode (WGM) microresonators, and microscaffolds for culturing microorganisms. The favorable properties of TPP, such as fine-tuning of the structure dimensions, the integration of the fabricated structures into a range of substrates, and its material flexibility, have pushed forward the design and potential of applications of WGM microresonators. Moreover, the material flexibility of TPP, along with its 3D capability and gentle processing conditions, has made it well suited for the fabrication of bioactive microscaffolds with spatially varying properties.

The further sections of the present review are organized as follows: In Section 2, we briefly describe the physical-chemical fundamentals underlying the TPP technique. We subsequently show the typical experimental setups and different materials employed in TPP. Section 3 provides an overview of the general applications of TPP. Emphasis is given to the fabrication of WGM microresonators and 3D microscaffolds to study microorganism’s development. Finally, Section 4 presents the overall conclusions and prospects of TPP in the near future.

## 2. Two-Photon Polymerization (TPP) Fundamentals

### 2.1. Two-Photon Absorption

**Figure 1:**
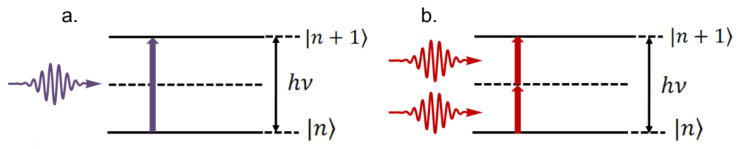
Energy diagram representing the absorption of (**a**) one and (**b**) two photons with the same energy to promote the electronic transition between the states |n⟩ e |n+1⟩. Reprinted with permission [40]. Licensed under a Creative Commons Attribution 4.0 International license.

The spatial confinement of TPP, which allows fabricating 3D microstructures with arbitrary geometry and submicrometric features, arises from the nonlinear nature of the two-photon absorption (TPA) phenomenon. The latter refers to an electronic transition excited by the combined action of two photons [2,36], such that the sum of the energy of the individual photons must be resonant with the transition energy [37]. When the photons have the same energy, as indicated in Figure 1b, the process is called degenerated [38]. TPA can also be observed with photons of different energies since the sum of their energies matches the energy gap.

In a quantum mechanics framework, TPA is described by considering the existence of a virtual intermediate state, which takes part in the electronic transition [36,39]. In this picture, a transition to a real state is possible only if the time interval between the two-photon is shorter than the lifetime of the virtual state [39]. Because the virtual state lifetime is extremely short, in the order of femtoseconds [37], TPA is usually described as the “simultaneous” absorption of two photons.

The TPA is a nonlinear optics phenomenon that can occur in the presence of high-intensity light, i.e., when the magnitude of the light electric field is comparable to the interatomic electric field ($E_{int}\approx 10^{11}\;V\cdot m^{-1}$) [36]. In this regime, the electric field induced in the material responds nonlinearly with the electric field of light, which can prompt unusual effects such as light generation at frequencies other than that of the incident light and absorption of light in the frequency range of transparency of the material [36]. When high-intensity light is employed, the absorption becomes dependent on the excitation intensity according to [36,38]:

(1) $$\alpha \left(I\right)=\alpha_{0}+\beta I,$$

in which, *α*_0_ and β are, respectively, the linear absorption and TPA coefficient, and I is the light intensity. β is directly associated with the TPA cross-section ($\sigma_{TPA}$) as stated in Equation (2) [38]:

(2) $$\beta =\frac{N\sigma_{TPA}}{h\nu }\;,$$

in which, N, h, and ν are the concentration of entities that constitute the material (e.g., atoms, molecules, etc.), the Planck constant, and the pump frequency, respectively. For most molecules, the TPA cross-section ($\sigma_{TPA}$) is in the order of $10^{-50}cm^{4}\cdot s\cdot photon^{-1}$ or 1 GM. Such unit is a reference to the Nobel-laureate physicist Maria Göppert–Mayer [38,41].

As light propagates throughout the material, its intensity is attenuated exponentially [37]. For light in the frequency range of transparency of the material, for which $\alpha_{0}=0$, the light dissipation rate along the propagation direction (z) is described by [37]:

(3) $$\frac{dI\left(z\right)}{dz}=-\beta I^{2}\left(z\right)\;.$$

**Figure 2:**
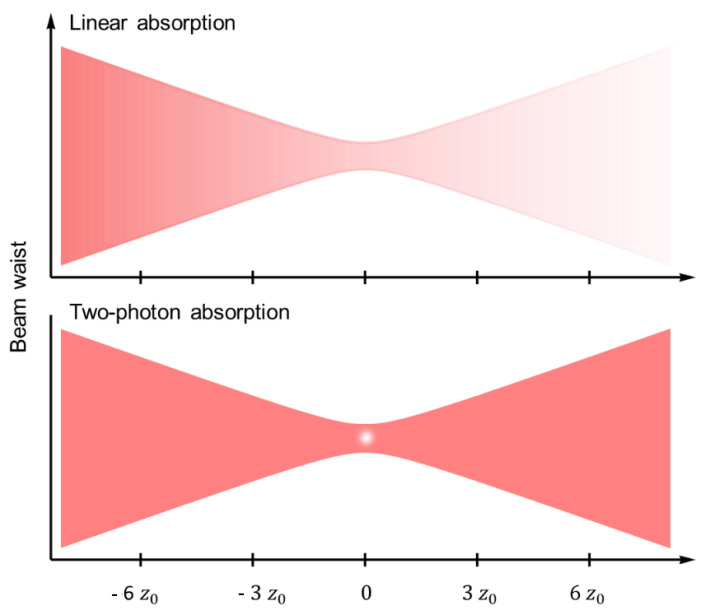
Attenuation of a focused laser beam prompted by the absorption of one (above) and two (below) photons. The white color indicates attenuation. The horizontal axis is given in units of the confocal parameter ($z_{0}$): the distance from the focal plane at which the beam waist increases by a factor of $\sqrt{2}$. Reprinted with permission [40]. Licensed under a Creative Commons Attribution 4.0 International license.

From Equation (3), we see that the dissipation rate for a TPA process depends quadratically on the light intensity. The nonlinear dependence of TPA on the intensity of light, together with the relatively small TPA cross-section of materials, allows attaining spatial confinement of the excitation when focused high-intensity lasers are used, such as Ti:sapphire lasers [38]. An illustration comparing the light dissipation produced by one and two photons is presented in Figure 2. For the linear absorption, the light intensity is exponentially attenuated as it propagates through the medium. In contrast, for the TPA case, attenuation is confined to the laser focal volume.

The spatial confinement of the excitation promoted by TPA allows to restrict the polymerization reaction to the focal volume of a high-intensity laser, which confers 3D capability and high resolution to the TPP technique [2]. Besides, the quadratic dependence of TPA on the light intensity pushes the resolution down by a factor of $\sqrt{2}$, which allows fabricating sub-diffraction features [42].

### 2.2. Two-Photon Polymerization (TPP)

Since the 1990s, TPP technique [3] has been widely used in the development of different technological devices due to a varied range of advantages. In this technique, the utilization of high-intensity pulse lasers allows the observation of nonlinear optical phenomena in the irradiated sample. Besides that, the polymerization of small volume (called voxels) [43] occurs only near the focal spot, giving the sculpted structure finer resolution (bellow to the diffraction limit) [9]. Moving the laser beam into the sample, it is possible to fabricate three-dimensional structures without shape limitation, with complex contours, including manipulable devices [9,44]. Samples employed in TPP usually are composed of monomers, hybrid sol-gels materials, hydrogels, or composite matrices [45,46]. These compounds are mixed into a photoinitiator, an organic molecule responsible for absorbing the laser energy and starting the polymerization procedure. The use of different monomers or elements in the employed sample allows matrices with specific features (Section 2.4), which can be important for desired applications.

**Figure 3:**
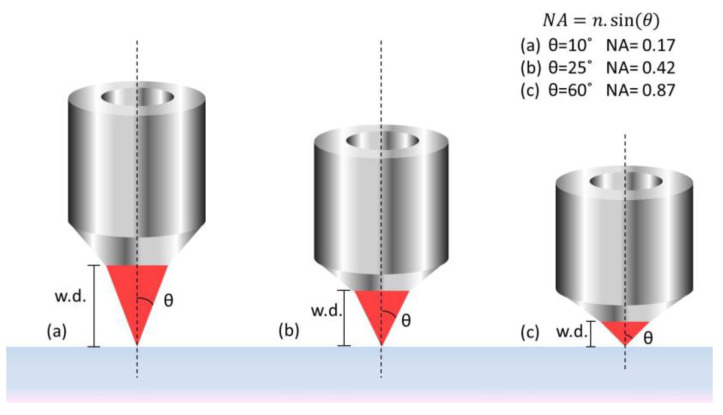
Light focusing through a microscope objective lens with different numerical aperture (NA) and working distance (w.d.). (**a**) NA = 0.17, (**b**) NA = 0.42 and (**c**) NA = 0.87. Reproduced with permission [38]. Licensed under a Creative Commons Attribution 4.0 International license.

An essential optical element to TPP technique is the microscope objective, which is used to focus the laser beam into the sample. Figure 3 shows a schematic diagram for different objectives [38].

The greater the numerical aperture, the smaller the working distance, i.e., the distance between the focal plane and the surface of the objective. The smallest spot size achieved is given by Abbe’s expression [47]:

(4) $$r=\frac{0.61\lambda }{NA}$$

in which, r is the focal spot radius, λ is the light wavelength, and NA is the numerical aperture of the microscope objective. The numerical aperture is defined by Abbe as:

(5) NA=n.sin(θ)

in which, n is the index of refraction in the focusing medium, and *θ* is the convergence angle of the beam.

However, most pulsed lasers have an approximately Gaussian beam profile. Besides that, two-photon polymerization setups use microscope objectives with high numerical aperture, and the minimum beam waist can be expressed by:

(6) $$w_{0}=\frac{\lambda }{\pi .NA}\sqrt{n^{2}-NA^{2}}$$

where $w_{0}$ is the beam waist at the focal plane. The beam waist is defined as the distance from the center of the Gaussian beam up to the point where the amplitude of the electric field falls to 1/e of its maximum.

**Figure 4:**
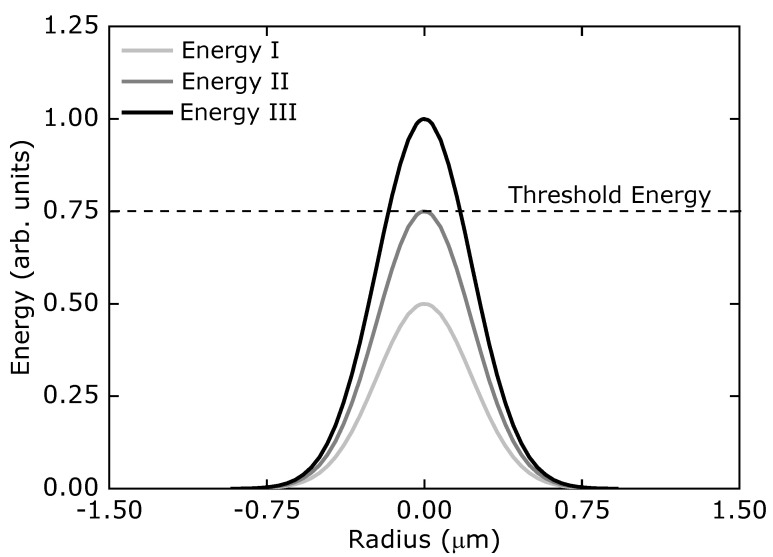
Threshold power relative to various Gaussian profiles. Polymerization is not observed if peak power is below the threshold power.

The photopolymerized structure resolution can also be improved when the fabrication procedure is performed around the polymerization threshold. When the laser beam using energy below the polymerization threshold value irradiates the sample, no polymerization occurs (Figure 4). The more the peak power gets closer to the threshold power, the smaller is the voxel size.

In the next section, we discuss the experimental features of TPP technique.

### 2.3. Experimental Aspects of TPP

**Figure 5:**
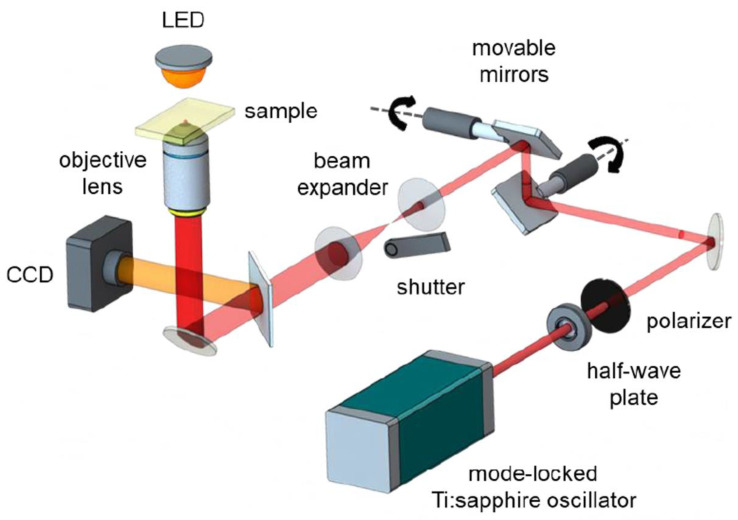
Representation of the femtosecond laser microfabrication via TPP setup, showing the basic components of the system. Reprinted with permission [40]. Licensed under a Creative Commons Attribution 4.0 International license.

The optical systems used for microfabrication via TPP vary in different aspects, including the laser system itself and the optical components. A schematic showing the basic components of a TPP system is presented in Figure 5. The laser beam comes from a mode-locked Ti:sapphire oscillator and is focused on the photoresist using an objective lens. The pulse energy can be adjusted by using a half-wave plate and a polarizer. A set of two movable mirrors is used to scan the laser beam transversally in the photoresist. A telescope is used to expand the laser beam to match the objective aperture. As previously mentioned, the choice of the objective lens is essential, as it determines the focal volume, allowing control of the size and thickness of the fabricated structures. A translational stage is used for the sample positioning, and a mechanical shutter controls the laser exposure. Control programs are used to manage the shutter, movable mirrors, translation stage, exposure time, and laser scanning speed. The combination of movable mirrors with the 3D translational stage allows the fabrication of the microstructures over a large area in the order of millimeters. The TPP writing process is performed in a successive layer approach and can be monitored in real-time with a CCD camera and a backlight illumination, while the refractive index of the polymerized structure is slightly changed during the process. After the TPP fabrication, the sample is immersed in an appropriate solvent, to remove the unpolymerized material.

### 2.4. Materials Used for TPP

In polymerization systems initiated by light absorption, there are two classes of photoresist materials that can be employed, and they are denominated as positive or negative photoresists. A great advantage of TPP technique is the maskless fabrication, due to the focalization of the laser beam into the sample. When exposed to light, positive photoresists, such as S1800 [48], become soluble and, in the non-irradiated region, the material solidifies. In the case of negative photoresists, such as SU-8 [49], the process is reversed, and the region that will be solidified is the one that is exposed to light.

A variety of materials can be used in the TPP experiments. The matrices used in this laser fabrication technique should be selected considering the application of interest. For instance, acrylate monomers are widely used in TPP [34,50]. These compounds usually present high optical quality and are biocompatible with different microorganisms [51,52]. Besides that, these resins can be easily functionalized with several materials, giving specific optical or biological features [53,54,55,56]. The combination of two acrylate monomers, for example, can improve the hardness and prevent the shrinking of the final structure [50]. Epoxy-based compounds, such as the SU-8, are also attractive for the TPP experiment, allowing the manufacture of structures with a high aspect ratio [57]. Hybrid materials also are noteworthy in TTP experiments [58,59]. These matrices combine the advantages of organic compounds (for instance, flexibility and chemical resistance) with those of inorganic components, improving different properties of the final device (such as mechanical stability and optical quality in several wavelengths) [60].

Negative-tone resins produced by Nanoscribe GmbH & Co. (Eggenstein-Leopoldshafen, Germany) also are applied in the TPP setups [61,62,63]. These resins, called IP Photoresins, can be used to fabricate several technological devices, such as optical elements for the biological scaffolds. Other commercial matrices, such as Ormocer^®^ and Ormocomp^®^, have been used to sculpt devices for photonics, optoelectronics, and biological areas [48,64,65]. However, many groups have developed their own samples, directing it to the desired application. For instance, some researchers have expended efforts to develop photocrosslinkable gelatin hydrogels for biofabrication applications [66,67,68,69,70]. For tissue engineering applications, photoreactive resin based on poly(trimethylenecarbonate) (PTMC) has been modified through chemical reactions, aiming at the fabrication of highly porous and biodegradable scaffolds [71].

Still, regarding the employed samples in the TPP experiments, photoinitiator compounds are essential to the technique. Many groups have been working on the development or testing of new photoinitiators for TPP experiments [72,73]. Usually, they look for compounds with a high value of TPA cross-section ($\sigma_{TPA}$).

After absorbing the laser pulse energy, radicals are generated. Then, these radicals can react with other sample components (monomers) and start the photopolymerization procedure. There are two mechanisms through which the photoinitiator can trigger the polymerization reaction in the TPP: polymerization by free radicals generation and cationic polymerization [74].

The active species in the polymerization by free radicals are neutral, and their incorporation with the monomers is not influenced by the polarity of the monomer or the solvent. However, molecular oxygen can inhibit its action, which is often advantageous for better resolutions in manufactured devices [9]. In free radical polymerization, photoinitiators can be classified into two groups, according to the mechanism of radical formation. The photoinitiators classified as Norish Type I, when absorbing one or two photons, are brought to an excited state and undergo breakdown (or chemical fission), producing radicals, in general, equally reactive. Among the Norish Type I photoinitiators, the commercial compound named Lucirin TPO-L [75] is a typical material employed in TPP fabrication. The photoinitiators classified as Norish Type II, for example, benzophenone [76], do not generate radicals independently, and the use of a co-initiator is necessary. Such photoinitiators undergo a bimolecular reaction with a co-initiator to create radicals. Thus, as the bond cleaves homolytically after excitation, two radicals are generated, and at least one of them will initiate the polymerization reaction [77].

In cationic photoinitiation systems, an acid is generated after laser irradiation. Unlike photopolymerization initiated by free radicals, sometimes cationic polymerization can occur without chain growth control. However, some photoinduced methods allow better control of the cationic polymerization [78]. Also, cationic photopolymerization is not inhibited by oxygen. Different classes of monomers, such as epoxy, vinyl esters, and siloxanes can be polymerized by a cationic mechanism.

The variety of materials that can be employed in the TPP experiments, added to the ease of sophisticated structure fabrication, makes this technique very attractive for developing the device. In the next section, we present some applications of the sculpted structures by means of TPP technique.

## 3. Devices Fabricated via TPP: General Applications

### 3.1. Functionalized Structures

**Figure 6:**
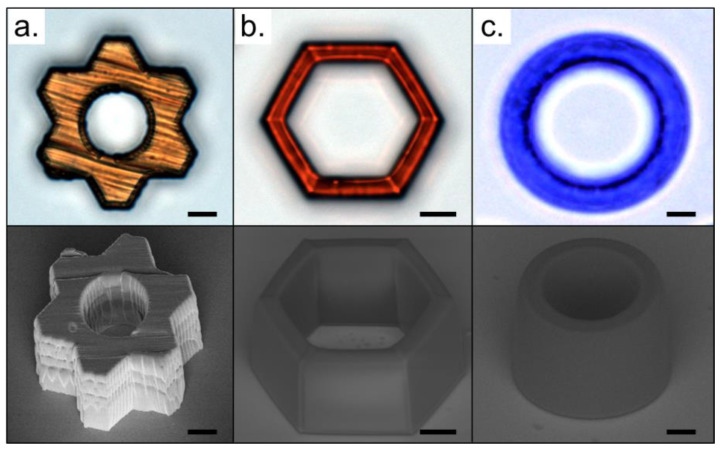
Optical microscopy (**top**) and SEM images (**bottom**) of 3D structures fabricated using azobenzene-based dyes as a photoinitiator in a concentration of 1.00 wt%: (**a**) Disperse Orange 3 (DO3), (**b**) Disperse Red 13 (DR13), and (**c**) Sudan Black B (SBB). The scale bar is 20 μm.

One of the most attractive advantages of the TPP technique is related to the production of functionalized devices, which can be applied in several technological fields. Usually, these devices are designed with specific properties according to the application need. There are different methods able to realize the device’s functionalization. The mixture of the functionalizing compound with the host sample (used in the TPP experiment) is the simplest method to achieving devices with special properties. For optics and photonics applications, organic dyes can easily be added to the pure matrices only by mixing the desired elements [53,55,79]. Several works were performed to manufacture microstructures doped with fluorescent dyes, such as Rhodamine B, Disodium Fluorescein, and Stilbene 420 [53]. Specifically, the use of these three dyes allows the fabrication of RGB-like syntonized devices. Other optically active compounds of considerable interest in photonics are the azobenzene-based dyes, such as Disperse Orange 1 (DO1), Disperse Red 13 (DR13), or Sudan Black B (SBB). This kind of dye can also be used as a photoinitiator in TPP experiments, keeping their original optical properties even after all microfabrication procedures [80]. Figure 6 shows some 3D structures fabricated using azobenzene-based dyes as a photoinitiator.

As can be seen in Figure 6, structures fabricated through the free radical generation in azoaromatic compounds present good structural integrity. The final structure color is similar to the pristine dye solved in ethanol (the solution used to incorporate the dye into the pure resin). Moreover, due to a high dye concentration used as a photoinitiator, a significant amount remains unchanged in the produced structures, and some optical properties, such as optically induced birefringence, can be monitored [80].

Polymeric materials sometimes have poor physical-chemical properties, which makes difficult their application in microdevices. An excellent alternative to solve this problem is, for example, the development of polymeric materials reinforced with carbon nanotubes, a material widely known to have interesting electrical, thermal, optical, and mechanical properties [81,82,83,84]. Even a low concentration of carbon nanotubes dispersed into the polymeric matrices (0.01 wt%) can significantly improve the device’s properties [85].

**Figure 7:**
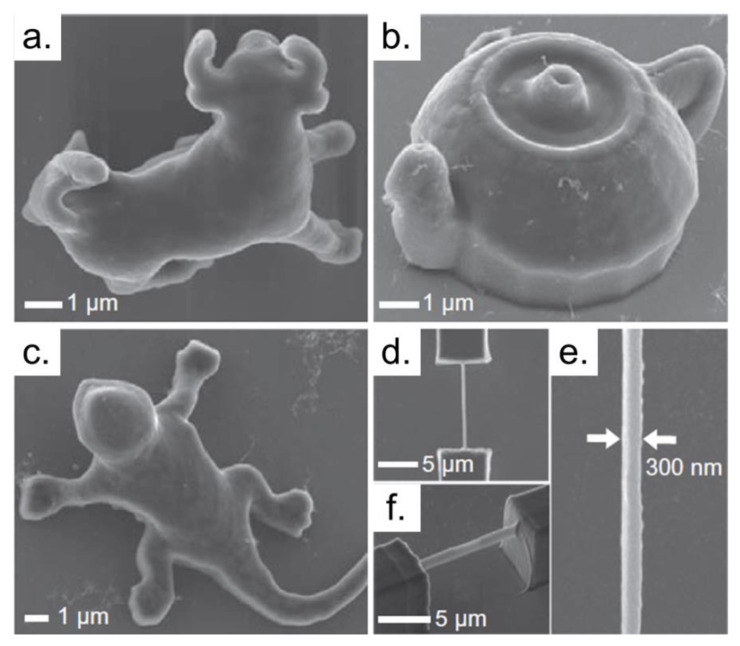
3D micro/nano structural SWCNT/polymer composites are fabricated by using the TPP lithography. The structures in (**a**–**f**) are a 8-lm-long micro bull, a micro tea pod, a micro lizard, a nanowire suspended between two micro boxes, magnified image of (**d**), and a perspective view of the nanowire, respectively. Reproduced with permission [87]. Copyright © 2013, Elsevier.

Some groups have performed the fabrication of 2D and 3D structures using polymeric matrices reinforced with single-walled or multi-walled carbon nanotubes (SWCNT or MWCNT) [85,86,87,88,89]. Figure 7 shows some examples of different structures fabricated via TPP, using SWCNT/polymer composites.

Still, regarding microstructures fabricated using SWCNT/polymer composites, researchers have demonstrated that the carbon nanotubes are uniformly distributed into the sample [85], and also, they can stay aligned inside the photopolymerized structures, according to the laser scanning direction [88,89]. Besides that, structures fabricated with MWCNT/polymer matrices, grown upon two pairs of Au electrodes, had different electrical conductivity responses according to the laser fabrication direction. For instance, structures fabricated with parallel scanning (in respect to the Au electrodes) were 1000 times more conductive than those fabricated with perpendicular scanning [89]. An increase in the electrical conductivity of over 11 orders of magnitude can be observed in acrylate-based devices functionalized with 0.2 wt% MWCNT [89].

Mechanical properties are also improved after CNT incorporation in acrylate resins. For structures reinforced with 1 wt% of SWCNT, the change of the modulus of elasticity and viscoelasticity is notable [85]. If the MWCNT concentration increases in the acrylate material, the shrinkage of the photopolymerized structures decreases [89].

**Figure 8:**
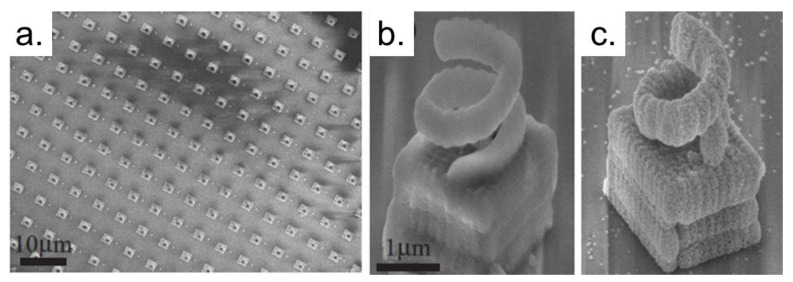
(**a**) 78 × 58 μm^2^ SEM image of a 3D periodic silver-coated structure fabricated on a hydrophobic coated glass surface. (**b**) Tilted magnified view of an individual uncoated polymer structure composed of a cube (2 μm in size) holding up a spring (height 2.2 μm, inner diameter 1 μm). (**c**) SEM image of an individual silver coated structure after electroless plating. Reproduced with permission [90]. Copyright © 2006, The Optical Society.

The device functionalization also can be performed after the complete laser fabrication procedure [65,90]. Figure 8 shows 3D metallic structures fabricated in a large area by TPP, using for this purpose a microlenses array that allows the fabrication of hundreds of devices simultaneously [90]. The structures’ metallization was realized by depositing thin films of small silver particles via electroless plating. Chemical changes of the resin and the hydrophobic coating of the glass substrate avoid metal deposition and confine the adhesion on the polymer [90].

**Figure 9:**
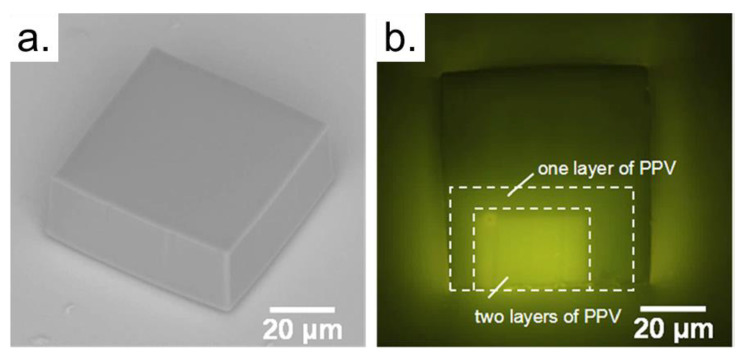
(**a**) SEM image of a polymeric microstructure fabricated by fs-laser writing via 2PP. (**b**) Fluorescence microscopy image of the microstructure shown after it was selectively coated with PPV via fs-LIFT. This image was obtained with excitation at 450–490 nm. Reproduced with permission [92]. Copyright © 2021, Springer Nature.

The use of TPP combined with other laser fabrication techniques, such as Laser-Induced Forward Transfer (LIFT), allows selective structure functionalization or even selective microorganisms inoculation [91]. Figure 9 shows a microstructure fabricated via TPP and selectively coated with a conducting polymer (PPV). It is important to highlight that the transferred polymer keeps its original features, even after the laser irradiation on the donor material. Besides, a material can be LIFTed several times upon the photopolymerized device [92], expanding its use to functionalize microstructures.

**Figure 10:**
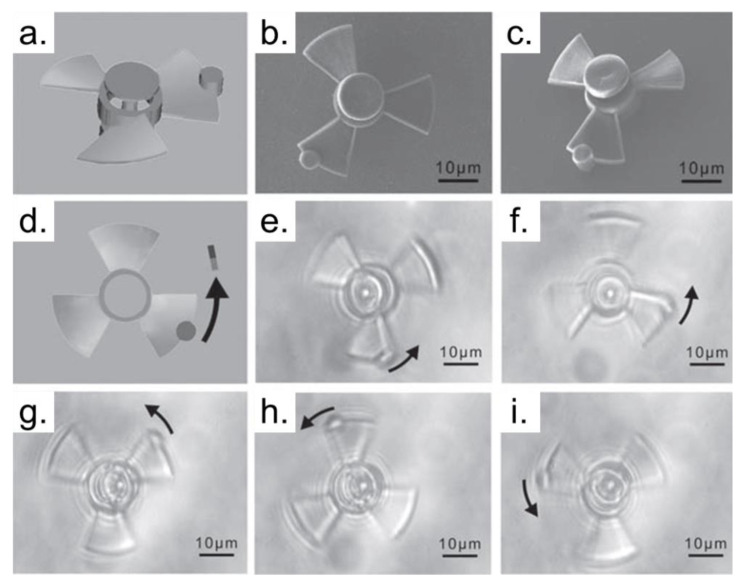
Remote control of the micro-turbine in acetone. (**a**) Model of the micro-turbine. (**b**) and (**c**) SEM images of the micro-turbine. (**d**) Top view scheme model for circumgyration. (**e**–**i**) Optical microscopy images of the microturbine in a circumgyration cycle. For remote control and observation of the micro-turbine, a piece of ferromagnet was placed on a vortical device around the objective lens. Reproduced with permission [93]. Copyright © 2010, John Wiley and Sons.

Structures that can be manipulable are very attractive to various fields, mainly for microfluidics or biomedical research [44,93,94]. The structure’s movement can be achieved through the use of optical tweezers or magnetic manipulation. An example of manipulable structures is presented in Figure 10.

In Figure 10, a photopolymerizable ferrofluid was developed, and 3D micro-turbines were fabricated via TPP. The micro-turbine size is approximately 35 μm in diameter with a central axletree and three blades [93]. These kinds of devices can be moved and controllable aided by an external magnet. Moreover, these devices can turn clockwise and anticlockwise. Based on these results, the importance of manipulable devices is evident, and is possible thanks to the TPP process.

Functionalized devices are essential for several applications. One of the most significant areas is the whispering gallery mode microresonators, which we discuss in the next section.

### 3.2. Whispering Gallery Mode Microresonators

Optical microresonators are microstructures that strongly confine light, both in the time and space domains [95]. They have been widely exploited for basic studies on the light–matter interaction [96,97], and for numerous applications, ranging from lasers to sensors [98,99,100]. In particular, optical microresonators supporting whispering gallery modes (WGM) are noteworthy due to their unique features, such as high temporal confinement of light, high sensitivity to the environment, and their ease integration in photonic systems [95]. A light beam can travel over 10^6^ roundtrips inside a WGM microresonator, which significantly enhances light–matter interactions and makes it possible to achieve a plethora of scientific discoveries and technological breakthroughs [101,102,103,104,105,106,107,108].

**Figure 11:**
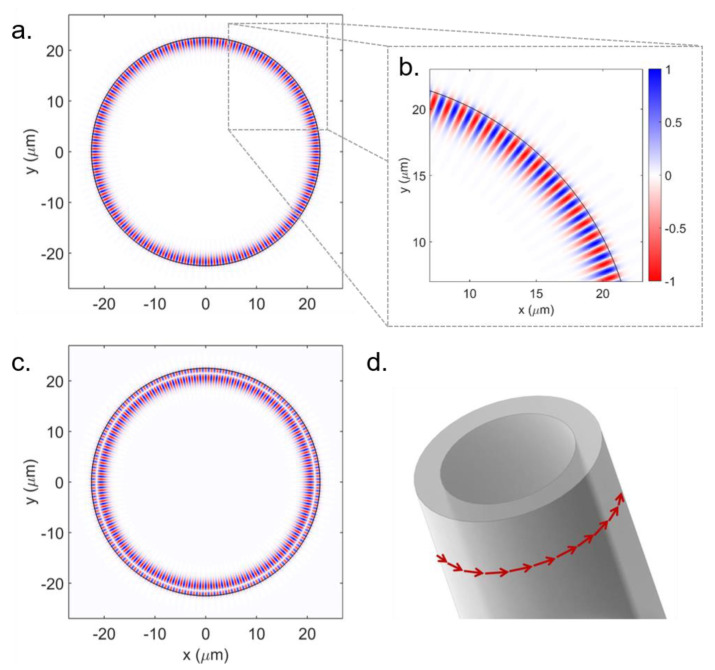
$E_{z}$ field amplitude over a transverse plane of the cylinder for the azimuthal order m=120 and the following radial orders: (**a**,**b**) *l* = 1 (*λ*_120,1_ = 1668.2 nm) and (**c**) *l* = 2 (*λ*_120,2_ = 1579 nm). Item (**b**) shows a zoomed-in view of the field distribution displayed in (**a**), along with the color bar for all the field distributions. The circumference corresponds to the cylinder’s edge. To obtain these field profiles, the refractive indices of both media and microcylinder’s radius were set to *n*_1_ = 1.51, *n*_2_ = 1.0 and *a* = 22.5 µm in Equation (9). (**d**) Geometric Optics picture of the WGMs propagating along the rim of a microcylinder. Adapted with permission [40]. Licensed under a Creative Commons Attribution 4.0 International license.

WGMs are electromagnetic waves that propagate along the rim of circular structures, e.g., cylinders and spheres, by total internal reflection (TIR) [109]. In a geometric optics framework, the WGMs can be seen as light rays that perform a close trajectory after a round trip in the circular-shaped structure, as shown in Figure 11d. The theoretical analysis of the WGMs in a given geometry is obtained by solving Maxwell’s equations with the proper boundary conditions [40,110]. To provide an insight on the theoretical aspects of WGM microresonators, we will take dielectric microstructures with cylindrical symmetry as an example. For this particular case, the WGMs are electromagnetic fields that propagate in the azimuthal direction and have a zero axial propagation factor [111].

The resonance takes place when the fields interfere constructively after a roundtrip in the structure, i.e., [40]:

(7) $$\vec{E}\left(\rho ,\phi \right)={\vec{E}}_{0}\left(\rho \right)e^{i\beta \phi }={\vec{E}}_{after\;a\;roundtrip}\left(\rho ,\phi \right)={\vec{E}}_{0}\left(\rho \right)e^{i\beta \left(\phi +2\pi \right)}.$$

∴ β=m;                             m=1,2,3,…

where (ρ,ϕ,z) are the radial, azimuthal, and longitudinal coordinates; ${\vec{E}}_{0}$ is the electric field amplitude; and β is the propagation constant in the azimuthal direction. The condition given by Equation (7) takes into account the selectivity of the fields allowed in the microstructure. The fields for which β is an integer constitute the modes of the structure.

However, there is a spectrum of modes for each polarization of the electromagnetic field, which are expressed as TM^(z)^ modes when the magnetic field in the longitudinal direction is null (*H_z_* = 0), and TE^(z)^ modes when the electric field in the longitudinal direction is null ($E_{z}=0$) [40]. For a microcylinder, the longitudinal component of the electric field for a TM^(z)^ mode is given by [110]:

(8) $$E_{z}\left(\rho ,\phi ,t\right)=\{\begin{array}{c} a_{m}\;J_{m}\left(k_{1}\rho \right)e^{i\left(m\phi -\omega t\right)},\;\rho <a.\\ b_{m}\;H_{m}^{\left(2\right)}\left(k_{2}\rho \right)e^{i\left(m\phi -\omega t\right)},\;\rho >a.\; \end{array}$$

for which: $k_{i}=n_{i}\frac{2\pi }{\lambda_{0}},\;i=1,2$.

In the Equation (8), a is the microcylinder’s radius, $J_{m}\left(k_{1}\rho \right)$ and $H_{m}^{\left(2\right)}\left(k_{2}\rho \right)$ are Bessel function of first kind and Hankel function of second kind, respectively, m is the azimuthal order, $a_{m}$ and $b_{m}$ are the field amplitude coefficients, $n_{i}$ is the refractive index of each medium, and $\lambda_{0}$ is the wavelength of light.

The boundary conditions of the tangential fields at the dielectric interface are applied to obtain the characteristic equation for the TM^(z)^ mode [110]:

(9) $$\frac{1}{n_{1}}\;\frac{J_{m}\left(k_{1}a\right)}{J_{m}^{’}\left(k_{1}a\right)}=\frac{1}{n_{2}}\frac{H_{m}^{\left(2\right)}\left(k_{2}a\right)}{H_{m}^{{\left(2\right)}^{’}}\left(k_{2}a\right)};\;k_{i}=n_{i}\;\frac{2\;\pi }{\lambda_{0}}\;\left(i=1,2\right).$$

For each azimuthal order (m), Equation (9) gives a set of complex wave numbers that constitute the TM^(z)^ modes of the cylinder. Their real part gives the resonant wave numbers, whereas their imaginary part accounts for radiation losses resulting from the cylinder’s curvature [40,110,111].

The total internal reflection imposes an additional boundary condition that constrains the allowed modes to the interval [110]:

(10) $$k_{2}a<m<k_{1}a,\;\mathrm{in}\;\mathrm{which}\;k_{i}=n_{i}\frac{2\pi }{\lambda_{0}}.$$

Thus, the WGMs are the roots of the characteristic equation (Equation (9)) that meet the TIR constraint.

As an example, in Figure 11a–c it is shown the $E_{z}$ distributions over the transverse plane of a microcylinder, obtained for the WGMs of azimuthal order m=120 and different radial orders (l=1,2). The azimuthal order corresponds to the number of field amplitude maxima along the azimuthal direction, while the radial order corresponds to the number of field amplitude maxima along the radial direction. As shown in Figure 11a, the field distribution of fundamental radial modes is more confined to the microcylinder’s surface. Albeit, higher-order radial modes (Figure 11c) exhibit field distributions spread out inside the resonator, making them more susceptible to losses and rendering lower peak-intensity. The electromagnetic field confined in the microcylinder exhibits an exponentially decaying tail that extends to the surrounding medium, with a decay length of a few hundred nanometers. This characteristic allows light coupling in and out of the structure, being exploited, for instance, in the development of sensors.

For fundamental radial WGM modes (m≫1,l=1), the resonance condition can be described by [110]:

(11) $$\lambda_{0}=\frac{2\;\pi }{m}\;n\;a$$

where $\lambda_{0}$ is the resonance wavelength, m is the azimuthal order, and n and a are the refractive index and the radius of the microresonator, respectively. Note that, since the fundamental radial WGM modes are strongly confined to the microcylinder’s surface, their effective index can be approximated to the microresonator’s refractive index ($n_{eff}\sim \;n$). Equation (11) is helpful to estimate the spectral position of the WGM resonances without solving the characteristic equation. From Equation (11), the free spectral range (FSR), which is the frequency/wavelength spacing between consecutive azimuthal modes, can be estimated by [98,112]:

(12) $$FSR=\frac{\lambda_{0}^{2}}{2\;\pi \;a\;n}\;,$$

One of the most important parameters used to measure the time confinement of microresonators is the quality factor (Q), which is given by the ratio between the total energy stored and the power dissipated in the resonator in one cycle of the field [112,113]:

(13) $$Q=\omega_{0}\frac{stored\;energy}{dissipated\;power\;}=\frac{\omega_{0}\tau }{2}=\frac{\nu_{0}}{\Delta \nu }\;\;,$$

where $\omega_{o}$ is the angular resonance frequency, τ is the time for the stored energy to decay by a factor of 1/e, and $\nu_{0}$ and Δν are, respectively, the resonance frequency and its corresponding linewidth.

The energy inside the microresonator dissipates through many routes, being intrinsic or external to the resonator. The overall losses can be written as [114]:

(14) $$Q^{-1}=Q_{in}^{-1}+Q_{ext}^{-1}=\left(Q_{rad}^{-1}+Q_{mat}^{-1}+Q_{surf}^{-1}\right)+Q_{ext}^{-1}\;,$$

in which, $Q_{in}^{-1}$ ($Q_{ext}^{-1}$) are the intrinsic (extrinsic/coupling) losses, and $Q_{rad}^{-1}$, $Q_{mat}^{-1}$ and $Q_{surf}^{-1}$ are the major contributions to the intrinsic losses due to radiation, material, and surface scattering losses, respectively. Moreover, power dissipation in the microresonator can also occur due to the presence of surface contaminants (e.g., water adsorption) [115,116], and through nonlinear processes, such as multiphoton absorption [117], prompted by the high intensities achieved in WGM microresonators. The overall Q-factor is limited by the dominant source of loss.

WGM microresonators with high Q are able to confine light for a relatively long time, such that high circulating intensities are achieved even from moderate excitation levels, because of the extremely small volume of WGMs in these structures [99]. WGM microresonators with Q in the range of $10^{3}–10^{6}$ are called high-Q, and those surpassing $10^{7}$ are named as ultra-high-Q [96].

The materials used for fabricating WGM microresonators are diverse and depend on the application and fabrication technique. In particular, polymeric microresonators have become increasingly competitive with microresonators made of other materials due to their structural flexibility, ease of processing and functionalization, and low cost [99]. Moreover, there are polymers with a wide range of refractive indices and with electro-optic properties available [118].

Various techniques have been used to fabricate WGM polymeric microresonators, including direct lithography patterning [118,119], molding from a master structure [120,121], surface-tension-based approach [122], solution-printing technique [123], and femtosecond laser writing via two-photon polymerization (TPP) [17,51]. In the past two decades, the latter technique has attracted significant interest due to its 3D fabrication capability, fine-tuning of the structure dimensions, and the integration of the fabricated structures into a range of substrates [2,9].

**Figure 12:**
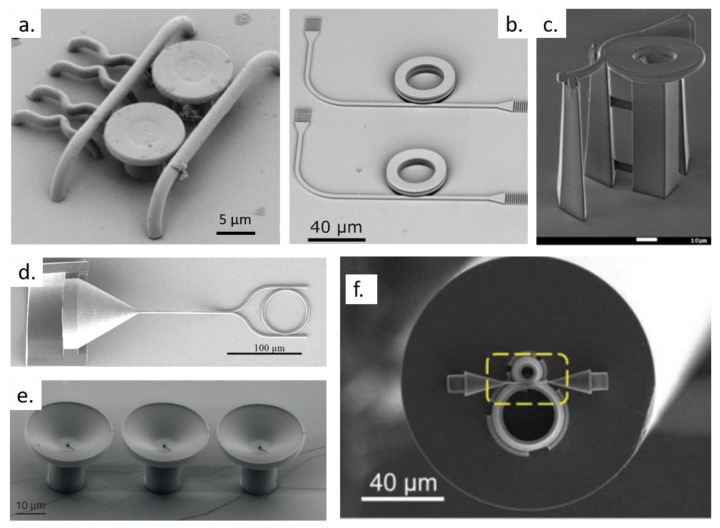
Polymeric microresonators fabricated by femtosecond laser writing via TPP. (**a**) microdisk resonators fabricated from a nanodiamond photoresist to enable quantum photonics applications, (**b**) two vertically-coupled microring resonators incorporated with liquid crystal networks and an azo dye to afford light-driven tunability, (**c**) microring resonator inside a glass microfluidic chip for biosensing, and (**d**) integrated microring resonator used for ultrasound detection. (**e**) Goblet-shaped microresonators fabricated on a PDMS elastomer substrate for coupling tunability, and (**f**) microring resonators fabricated on the facet of a multicore optical fiber for vapor sensing. Figure 12a Reproduced with permission [135]. Licensed under a Creative Commons Attribution 4.0 International license. (**b**) Reproduced with permission [131]. Copyright © 2018, American Chemical Society. (**c**) Reproduced with permission [132]. Copyright © 21019, The Royal Society of Chemistry. (**d**) Reproduced with permission [129]. Copyright © 2017, The Optical Society. (**e**) Reproduced with permission [133]. Licensed under a Creative Commons Attribution 4.0 International license. (**f**) Reproduced with permission [26]. Copyright © 2019, John Wiley and Sons.

Polymeric microresonators with several geometries have been fabricated by TPP, including toroids [124], disks [125,126,127], cylinders [17,128], rings [26,51,129], and spheres [130]. Moreover, the flexibility of geometry afforded by this technique allows the fabrication of suspended parts (Figure 12a,c), varying the height of structures on the same chip (Figure 12b), and wire bonding fiber-to-chip coupling elements (Figure 12d and Ref. [18]), which opens up new degrees of freedom for microresonator designing. For example, the microresonators displayed in Figure 12b, which were incorporated with a photoresponsive deformable material, are vertically coupled to integrated waveguides [131]. Owing to the 3D integration scheme, the microresonator spectral response can be dynamically tuned not only through its refractive index variation but also through its mechanical deformation by a remote optical stimulus. Another example is the integrated microresonator displayed in Figure 12d, which is coupled to a fiber coupler through a photonic bonding wire [129]. In addition to compactness, this architecture avoids alignment difficulties and reduces the losses related to fiber-to-chip coupling [18,29].

The integration of the microresonators into different substrates represents a great advantage of TPP over the other techniques. Figure 12c,e,f respectively shows microresonators fabricated inside a microfluidic channel, onto a silicone-based substrate, and onto the polished facet of a multicore optical fiber. The interface between microresonator systems and microfluidic channels/optical fibers gives rise to portable and easy-to-handle sensing units, which are promising tools for medical care, chemical manufacturing, and environmental monitoring [26,132]. Besides, the integration of microresonators into multicore fibers allows light coupling into and out through the different fiber cores, thus making the microresonator interface with laboratory instrumentation very convenient. The use of silicone as a substrate can enable various photonic applications by allowing reversible and precise tuning of the gap distance between pairs of microresonators through mechanical stretching [133,134]. It has been shown that this method affords a tuning precision comparable to a nano-positioning piezo stage but with the significant advantage that the microresonators are on the same substrate [133].

Another convenience of the TPP technique for the fabrication of microresonators is its material flexibility. Several polymeric formulations have been employed as photoresists, such as acrylic [51,135] and epoxy resins [130,132], and various materials have been incorporated into them to meet specific applications. Polymeric microresonators fabricated by TPP have been incorporated with organic dyes to realize optically active microdevices [125,130,134], with liquid crystal networks to confer elasticity for optical tunability [131,136], and with nanodiamonds to enable quantum photonics applications [135], among other materials [137].

WGM microresonators fabricated by TPP can achieve Q-factors, in the near-infrared, from 10^2^ until 10^6^) [124,125,138,139,140]. Since the WGMs are strongly confined to the microresonator’s edge, the Q-factor in such structures is primarily limited by light scattering at the microresonator surface. Therefore, the major challenge behind accomplishing a high-Q microresonator by TPP lies in ensuring optically smooth surfaces. As described in Section 2, the laser writing process gives rise to polymerized volumes (voxels), which overlap to create the geometrical shapes that constitute the microstructure. In order to produce microstructures with smooth surfaces, the exposure and laser scanning parameters must be adjusted to ensure that the voxels are properly overlapped, i.e., the surface roughness associated with the individual characteristics of the voxels is significantly reduced.

**Figure 13:**
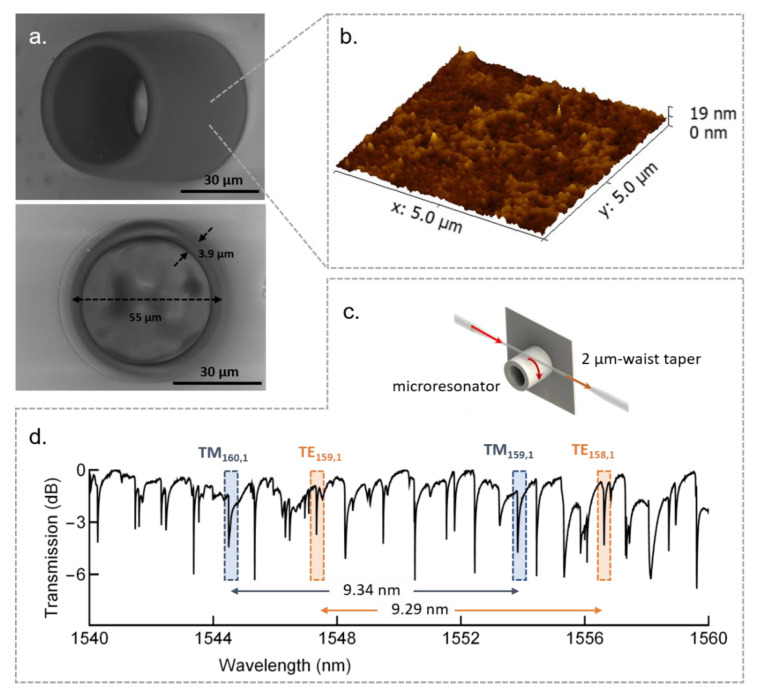
Polymeric microcylinder resonator fabricated by TPP. (**a**) Scanning electron micrographs of the microresonator. (**b**) Atomic force microscopy of the microresonator outer sidewall surface. (**c**) Schematics of the evanescent light coupling to the microresonators through the use of tapered fibers. (**d**) Spectral response of the microresonator. The attenuation peaks represent the WGMs accounting for different azimuthal and radial orders. Highlighted in the figure are the TE and TM fundamental radial WGMs, with their corresponding FSR. Adapted with permission [17]. Copyright © 2017, John Wiley and Sons.

Figure 13a shows one of the highest Q-factor WGM microresonators fabricated by TPP thus far [17]. It is a 22.5-µm-radius hollow microcylinder fabricated from an acrylate polymer using a 0.25 NA objective lens, 0.1 nJ pulses from a Ti:sapphire oscillator, and 40 µm/s of laser scan speed. Such parameters resulted in a longitudinal and transversal voxel overlap of 85% and 90%, respectively, translating into an average surface roughness as small as 2 nm. A morphology chart of the microresonator sidewall surface is shown in Figure 13b. The microresonator was interrogated by evanescent coupling through a 2 µm-waist tapered optical fiber, as depicted in Figure 13c. Its broadband spectral response around 1550 nm (Figure 13d) contains resonances accounting for different polarization states and different azimuthal and radial order WGMs. The TE and TM fundamental radial order WGMs and their corresponding FSR are highlighted in Figure 13d. The microresonator loaded Q-factor was found to be $1\times 10^{5}$, which is very close to the fundamental limit imposed by the acrylate material losses ($Q_{mat}=1.9\times 10^{5}$). Such Q-factor is competitive with the current semiconductor platforms and holds great prospects for several applications in photonics.

The microresonators with cylindrical geometry do not support light confinement in the vertical direction, which brings alignment difficulties to the coupling and leads to a more spread out mode volume. However, it can be challenging to accomplish more confined geometries, such as spheres, toroids, or even disks and integrated microrings, with smooth surfaces by TPP. Indeed, the Q-factor of microresonators with spherical [130] and toroid-like [124] geometries is in the order of $10^{4}$ or lower, and the Q-factor of integrated microrings is in the range $10^{2}-10^{3}$ [51,129,138,141]. Microdisks and integrated microrings suffer from additional scattering losses at their top surface, which is usually rougher than their sidewall surface due to the pointed shape of the voxel tips. One of the strategies used to reduce light scattering in a microdisk consists of designing its edge in the shape of a wedge and engineering the wedge angle to minimize the mode overlap with the top and sidewall surfaces [139].

One of the most studied applications of microresonators fabricated by TPP is lasing. The unique combination of Q-factor and tight mode confinement provided by WGM microresonators enables strong interaction between the light and the gain medium, which makes it possible to achieve low threshold and narrow linewidth microlasers [99]. Moreover, its small size and the possibility of on-chip integration make them promising as bright sources for integrated photonics applications.

**Figure 14:**
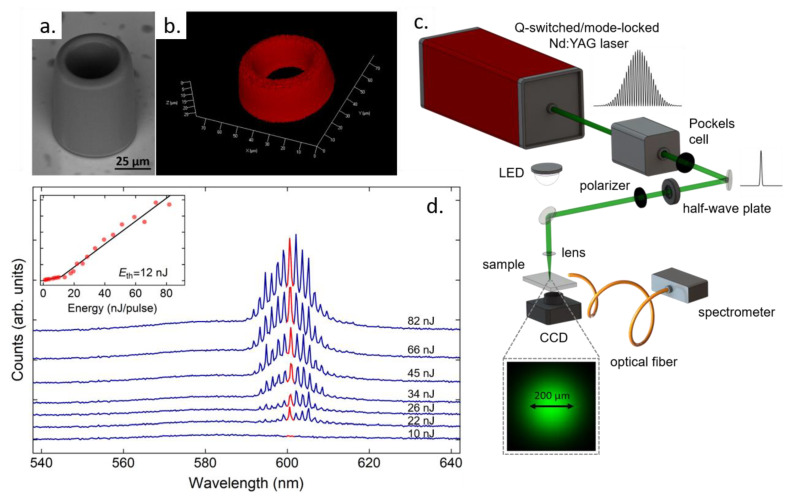
Rhodamine B-doped WGM polymeric microlaser fabricated by TPP. (**a**) Scanning electron, and (**b**) 3D reconstructed confocal fluorescence micrographs of the microlaser. (**c**) Setup used to excite the microlasers and collect their emission. (**d**) Emission spectra of a microlaser for the excitation energy levels indicated on the right hand side of the graph. To make the visualization easier, an offset was applied to the y-axis of each curve. The first curve represents the spectrum below lasing threshold. The inset shows a graph of the amplitude of the peak highlighted in red as a function of excitation energy. The graph is plotted on a linear scale. The threshold energy, which was obtained by fitting the experimental data with a bilinear curve (black line), is indicated in the graph. Adapted with permission [31]. Licensed under a Creative Commons Attribution 4.0 International license.

To realize microlasers, the microresonators are incorporated with organic dyes, such as Rhodamine B (RhB) [125] and Pyrromethene 597 [134]. The microresonators displayed in Figure 14a were fabricated from an acrylic-based photoresist incorporated with RhB [31]. Their hollow cylindrical geometry was strategically chosen since it supports a smaller number of high-order radial WGMs that would otherwise compete with the most fundamental WGMs for the pump energy. A 3D reconstructed confocal fluorescence micrograph confirmed the homogeneity of the dye distribution throughout the microresonator (Figure 14b), and absorbance/fluorescence analyses showed the neutrality of the polymeric matrix, i.e., it was shown not to affect the emission properties of the dye significantly.

The setup used to pump and collect the emission of the RhB-doped microlasers is shown in Figure 14c. In this setup, 100 ps-pulses from a Q-switched and mode-locked Nd:YAG laser centered at 532 nm are loosely focused on the microresonator top surface, and the microresonator emission is collected with a multimode optical fiber connected to a 250-pm-resolution spectrometer. Most of the other polymeric microlasers are also pumped with pulsed lasers with a time duration in the order of 10−100 ps [125,127,142]. The pulsed excitation prevents population transfer to the triplet state of the dye molecules, which would hinder its fluorescence quantum yield [143]. Overall, since the fluorescence lifetime of organic dyes is in the order of nanoseconds [144], the excitation being performed in the ps regime leads to less molecular cycles, thus extending the device lifetime.

The emission spectra of the RhB-doped microlasers feature a set of well-defined peaks within a narrow spectral range around 600 nm. The lasing threshold was measured at 12 nJ of pulse energy, which is well within the energy levels afforded by conventional ps-lasers. Besides, such a low lasing threshold is comparable to that which has been achieved for polymeric microlasers fabricated by other techniques [123,145,146], thus reinforcing the potential of TPP in fabricating active microresonators.

Some strategies have been developed to improve directionality, monochromaticity, and tunability for WGM microlasers fabricated by TPP. One of the strategies that has been used to overcome the lack of directionality of such microlasers consists in breaking their rotational symmetry. This can be accomplished by designing a spiral-shaped microdisk, which has been shown to exhibit directional lasing emission with a far-field divergence of about 20°−40° [142,147]. To ensure single-mode operation without significantly degrading the microlaser Q-factor, coupled WGM microlasers have been employed. It has been demonstrated that the coupling of two microdisks with different diameters suppresses some resonances and increases the effective FSR, which allows accommodating only one mode within the gain region [142,148]. Laser tunability has been achieved by using elastomer-based materials either as the substrate [134] or integrated into the microlasers [136]. For example, it has been reported that the lasing modes of a WGM microlaser integrated with photo-responsive liquid crystal elastomers reversibly reach a 0.51 nm- shift for every increase in the pump energy by 0.14 mJ/pulse.

**Figure 15:**
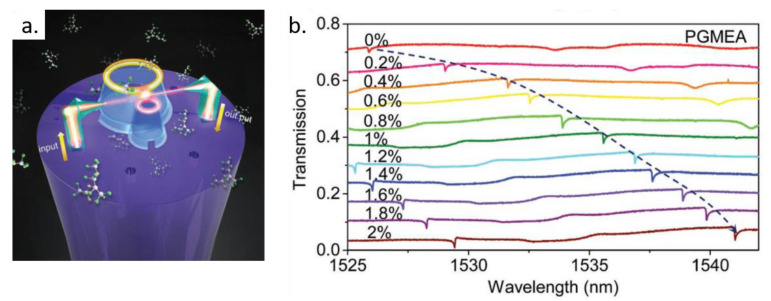
Microring resonators fabricated by TPP on the facet of a multicore optical fiber for vapor sensing. (**a**) Illustration of the light propagation in the photonic setup. (**b**) Spectral response of the microresonator when the fiber tip is placed in the vapor environment produced by an aqueous solution of propylene glycol monomethyl ether acetate (PGMEA) with the concentration varying from 0% to 2%, in steps of 0.2%. Adapted with permission [26]. Copyright © 2019, John Wiley and Sons.

Another important application of WGM microresonators fabricated by TPP is in sensor technology. The operational principle of WGM sensors is to monitor changes in their spectral properties prompted by environmental changes. Sensing of refractive index [128], temperature [141], volatile organic compounds [26], ultrasound waves [129], and the label-free detection of biological specimens [132] have been accomplished with these structures. The sensing mechanism usually relies on resonance wavelength shifts and/or resonance linewidth variations. An example of WGM sensor fabricated by TPP is shown in Figure 15. The multicore fiber containing the WGM polymer microresonator (Figure 15a) is placed in a vapor environment produced by an aqueous solution of propylene glycol monomethyl ether acetate (PGMEA) [26]. As shown in Figure 15b, by increasing the concentration of PGMEA, the WGMs are shifted towards longer wavelengths in response to microresonator swelling and refractive index variation of its surrounded medium. Besides PGMEA, the WGM sensor showed in Figure 15a was used to detect isopropanol and alcohol vapors, exhibiting a sensitivity of 21.7, 3.38, and 3.87 pm/ppm for each of these vapors in the low concentration range [26].

In the context of WGM sensors, it is desirable to have a clean resonance spectrum to prevent distortions associated with the overlap of multiple resonance peaks. One proposed solution to achieve mode cleaning is by incorporating a lossy element in a low concentration into the microresonator. It was shown that the incorporation of graphene oxide (GO) to WGM microresonators led to the suppression of a number of resonances in the spectrum without significantly reducing the Q-factor of the most prominent resonances [137]. Another route to mode cleaning is by decreasing the microresonator diameter to a few microns [149]. In this case, radiation losses would be considerably raised for all the modes, but the higher-order radial WGMs would be the most affected and ultimately the ones to be filtered out. The most loss-free strategy towards mode cleaning consists of reducing the sidewall thickness of hollow microcylinders or microrings to dimensions comparable to light wavelengths [121,149]. Such thin sidewall microresonators would only support the fundamental radial WGMs. However, it can be challenging to achieve, by the TPP technique, microresonators with such a small sidewall thickness that still maintain their structural integrity.

### 3.3. Three-Dimensional Scaffolds for Biological Investigations

**Figure 16:**
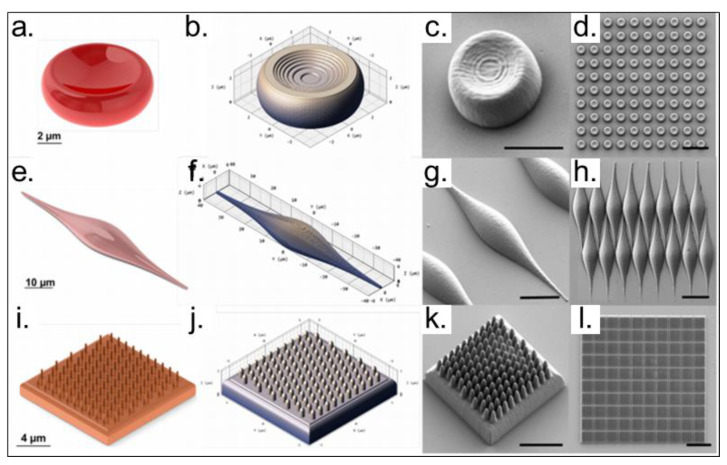
Images showing the designed and fabricated cell scaffolds using additive manufacturing with optimized parameters. (**a**–**d**) Red blood cell (RBC) model. (**e**–**h**) Smooth muscle cell (SMC) model. (**i**–**l**) Ciliated columnar epithelial cell (CEC) model. (**a**,**e**,**i**)—Rendered images of the 3D designs for a single cell. (**b**,**f**,**j**)—preview of a single cell generated in the DeScribe software after defining the parameters for the additive manufacturing fabrication process. (**c**,**g**,**k**)—Scanning electron micrographs of the fabricated individual cells, acquired using a 30° stage tilt, scale bar is 5 μm. (**d**,**h**,**l**)—120 × 120 μm^2^ cell arrays, scale bar is 25 μm. Reproduced with permission [152]. Licensed under a Creative Commons Attribution 4.0 International license.

At present, a significant part of the TPP sculpted devices is dedicated to biomedical applications due to the free form fabrication achieved by this technique, combined with ease functionalization of the microenvironment. The design and fabrication of biological scaffolds can be optimized to obtain platforms with biomimetic shapes and responses. Several researchers have investigated the geometry (or porosity) influence of photopolymerized scaffolds in the microorganisms’ development [21,150,151]. Besides, the mechanical and chemical resistance are also important features that should be characterized before applying these scaffolds in biological studies. Usually, to optimize the scaffold production, many parameters have been defined, and computational simulation is required. Figure 16 shows an example of three types of human cells profiles, red blood cell—Figure 16a–d, smooth muscle cell (SMC)—Figure 16e–h, and ciliated columnar epithelial cell (CEC)–Figure 16h–l, which were produced by TPP technique, with a negative-tone commercial acrylic photoresist from Nanoscribe [152].

The growing interest in the tissue engineering and regenerative medicine fields has motivated the development of several bio-applicable materials. For instance, the use of a highly reactive photopolymer in TPP experiments, with their fabrication parameters optimized, allows the production of scaffolds with large size (in the order of millimeters in the three dimensions), with relevant size for clinical application [71].

**Figure 17:**
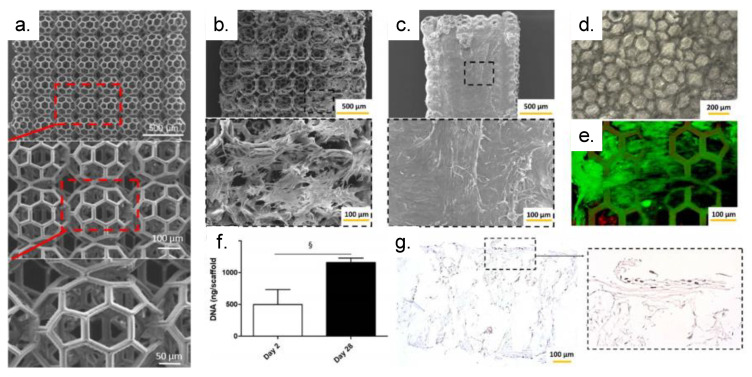
TPP-produced PTMC-based scaffolds support cell invasion and proliferation: (**a**) SEM images of the tri-layered PTMC-based scaffold at various magnifications (×50, ×150 and ×300). SEM images of cell-seeded scaffolds day 2 (**b**) and day 28 (**c**) post seeding. (**d**) Bright field and (**e**) live/dead illustrations of the colonized scaffold at day 28. (**f**) Cellular proliferation quantified by DNA assay (§ indicates statistical significance) and (**g**) Ki67 immunostaining (nuclei of positive proliferative cells are stained in black). Adapted with permission [71]. Licensed under a Creative Commons Attribution 4.0 International license.

Scaffolds sculpted from a biodegradable and biocompatible poly(trimethylene-carbonate) (PTMC)-based material by TPP (Figure 17a) supported the adhesion and proliferation of human adipose-derived stem cells (hASCs) [71]. Two days post-seeding, hASCs attached to the scaffold exhibited elongated filopodia (Figure 17b). Over 28 days of culture, the hASCs colonized the entire scaffold and formed a dense cellular layer on the surface of the scaffold (Figure 17c,d).

Figure 17e presents live/dead staining conducted after 28 days, showing a high cellular viability. The proliferation of the hASCs was validated by the significant increase of DNA (Figure 17f) and the presence of ki67-positive cells (Figure 17g) [71].

**Figure 18:**
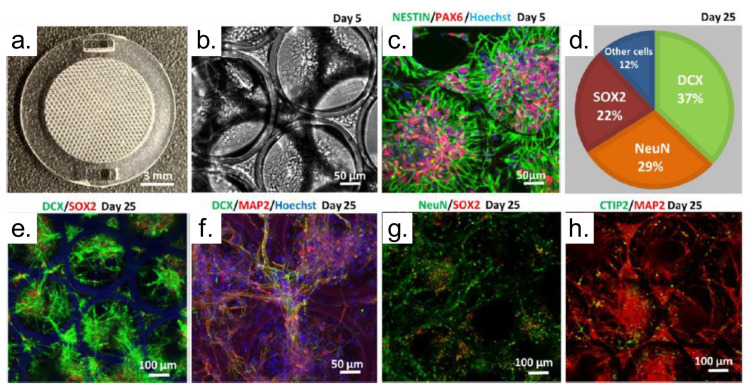
Generation of a 3D neuronal cell culture using laser-fabricated scaffolds. (**a**) General view of the scaffold structure (″honeycomb″) used for 3D neuronal culture development. (**b**) Phase-contrast image showing uniformly distributed cells within the scaffold 5 days after seeding. (**c**) Immunofluorescence image depicting well-accommodated NSCs in the pores of the scaffold. The labeled proteins are Nestin and PAX6, characteristic of cortical stem cells. (**d**) Quantitative ratio of young (DCX+) and mature (NeuN+) neurons as well as SOX2+ multipotent neural progenitor cells in the scaffold at day 25 of culture/differentiation. (**e**–**g**) Expression of young and mature neuronal markers DCX/MAP2/NeuN and SOX2, indicating a healthy formation of a neuronal network. (**h**) Differentiation of early-born CTIP2+ cortical neurons from neural stem cells. Reproduced with permission [153]. Copyright © 2021, American Chemical Society.

Cultured neuronal networks are the subject of many biological studies because they allow researchers to investigate neuronal activity in a controlled environment, in comparison to in a live organism. These experiments provide, for instance, a better understanding of the primary disease mechanisms and investigating the response to medicines [153]. TPP can be employed to produce 3D scaffolds, with high-resolution, using biocompatible and nondegradable photoreactive resins [153]. Figure 18 shows examples of 3D scaffolds fabricated using Dental LT Clear (DClear) resin employed in 3D neural cell culture and their characterization.

Figure 18a presents a 3D honeycomb-like scaffold fabricated via TPP, while Figure 18b shows a phase-contrast image demonstrating uniformly distributed cells within the scaffold 5 days after seeding. Still, in Figure 18b the scaffold design allows a noninvasive high-resolution visualization of real-time cell growth and neuronal network formation in a 3D environment. Immunofluorescence analysis showed that the fabricated scaffold provides a suitable 3D environment for the rapid development of neural stem cells (NSCs), facilitating the growth and long-term viability of neuronal networks (Figure 18c) [153], which may be related to the increased cell density in the cylindrical areas of the scaffolds that leads to strengthened neurons communications. Figure 18d represents a quantitative ratio of young (DCX+) and mature (NeuN+) neurons as well as SOX2+ multipotent neural progenitor cells in the scaffold at day 25 of culture/differentiation. In Figure 18, the expression of young and mature neuronal markers DCX/MAP2/NeuN and SOX2 indicate a healthy formation of a neuronal network. Finally, on day 25 of 3D culture, a large proportion of neurons already expressed the early-born cortical neuronal marker CTIP2 (Figure 18h) [153].

Microenvironments functionalized with biological compounds can also be sculpted using TPP. To evaluate the *E. coli* bacteria development, microenvironments were developed with selective functionalization using ciprofloxacin antibiotic. The inhibition of bacterial growth was primarily observed around the doped element. By analyzing images obtained from the microenvironment, it was possible to determine the maximum range of inhibition (12 μm), as well as to quantitatively determine the bacterial density in the inhibition halo [56].

**Figure 19:**
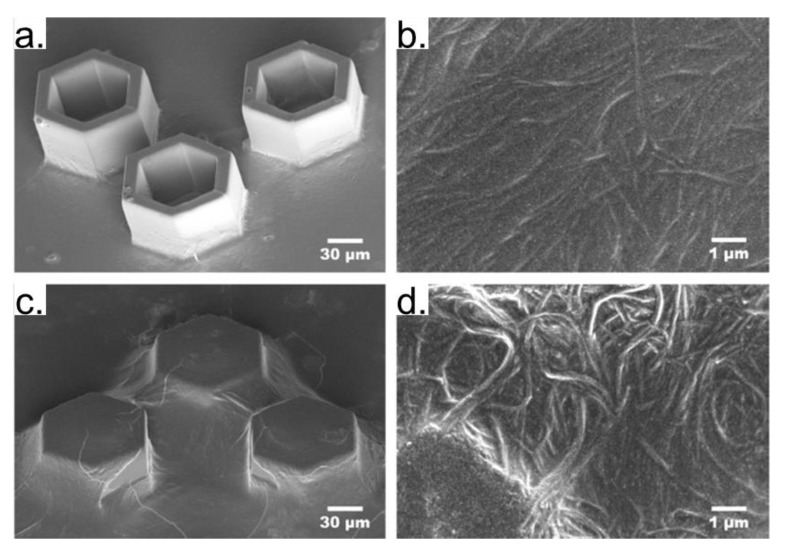
Bacterial cellulose (BC) grown on polymeric materials. (**a**) BC film formed after 6 h of bacteria incubation and (**b**) SEM image of the BC network grown in these structures. (**c**) BC film formed after 24 h of bacteria incubation and (**d**) SEM image of the BC network grown in these structures. Reproduced with permission [156]. Licensed under a Creative Commons Attribution 4.0 International license.

The natural biopolymer named bacterial cellulose (BC) is an attractive material for biomedical experiments because it presents uniform structure and morphology, besides unique features, such as high purity, good mechanical properties, chemical stability, and others [154,155]. This polymer is completely biocompatible and can be produced in practically any shape due to its high moldability. Figure 19 shows 3D acrylate-based structures fabricated via TPP used to evaluate the BC formation through the development of *Komagataeibacter xylinus* bacteria [156]. In Figure 19, it is possible to observe the biofilms when bacteria are inoculated in the microstructure for 6 h (Figure 19a) and 24 h (Figure 19c). As displayed in Figure 19a, even after a short incubation time, it is possible to observe a network of nanofibers around the structures. After 24 h, it can be seen a thin film covering the microstructures. Here, it is important to highlight that the BC grown in microenvironments, such as the one presented in the Figure 19, have the same chemical composition as BC grown in macroscale. The use of BC as a flexible substrate in TPP experiments is very interesting for the fabrication of versatile devices. The natural polymer was not altered by the laser irradiation, such that its original properties were kept until the end of the photopolymerization. The structures well adhered to the natural substrate, even after washing the sample in heated ethanol (a procedure required to remove the unpolymerized resin). BC flexible substrates are potential platforms for liquid diffusion mechanisms in microenvironments, opening, for example, novel approaches for drug delivery and tissue engineering.

**Figure 20:**
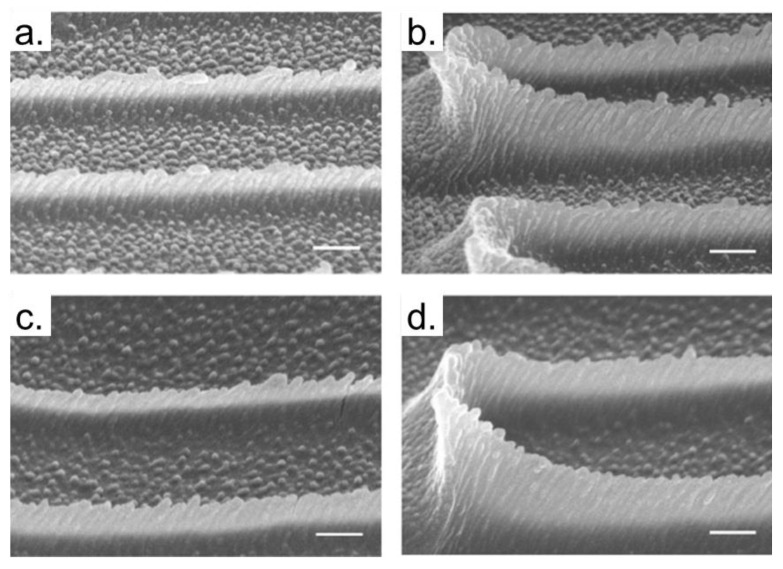
Replication of nanograss. (**a**,**b**) SEM images of the master nanograss. (**c**,**d**) nanograss replica. Scale bars, 200 nm. Reproduced with permission [157]. Licensed under a Creative Commons Attribution 4.0 International license.

Often, biological studies require several simultaneous analyses, and the TPP experiments (in their most straightforward setup) are not attractive to the microenvironments fabrication. However, the TPP technique can be used as a tool to create the main environment, which can be replicated several times later. As an example, Figure 20a,b shows SEM images of the master nanograss and Figure 20c,d nanograss replica [157].

The mass production of nanotopographic surfaces opens new possibilities to study the mechanism related to cell migration and development. Besides, several replica experiments provide more accurate results, which can ease the understanding of microorganisms growth.

Based on the few examples presented in this review, it is possible to see the substantial importance of TPP technique to device manufacturing. The versatility of this fabrication method allows several advantages for sophisticated technological applications.

## 4. Final Remarks and Perspectives

This review discussed the technological potential of the laser micromachining method known as Two-Photon Polymerization (TPP), which has a unique set of advantages. Several materials have been used in TPP experiments, such as polymeric matrices, composites, and gelatins. The search for new materials that can be used in this technique is one of the major efforts of different current researches. Structures fabricated by this technique have great applicability in optics, photonics, and biology, mainly due to the possibility of functionalization with several compounds of interest or the manipulable devices fabrication. Combining TPP technique with other laser-processing methodologies, such as Laser Induced Forward Transfer (LIFT), allows the selective functionalization of the sculpted structures, creating new approaches to functionalize the devices. Even living microorganisms can be transferred for 3D scaffolds, without any damage. The wide variety of 3D structures that can be fabricated using TPP is one of the attractive advantages of this manufacturing technique. The growth of microdevices manufactured via TPP is notable, principally regarding photonics devices and bio-platforms development. For instance, high-Q WGM microresonators with distinct geometries can be fabricated by TPP in few steps, achieving performance comparable to resonators fabricated using other fabrication methods. Bioscaffolds also can be sculpted using TPP and replicated several times, keeping their original features. Many microenvironments have been created for studies and monitoring of microorganisms development and can be ease functionalized with biological compounds of interest. Certainly, TPP is a consolidated laser fabrication technique with large applicability in the most diverse technological fields and will continue to be employed in sophisticated devices fabrication.

## Data Availability

The study did not report any new data.

## References

[1] Malinauskas M., Žukauskas A., Hasegawa S., Hayasaki Y., Mizeikis V., Buividas R., Juodkazis S. (2016). Ultrafast laser processing of materials: From science to industry. Light Sci. Appl..

[2] LaFratta C.N., Fourkas J.T., Baldacchini T., Farrer R.A. (2007). Multiphoton fabrication. Angew. Chemie Int. Ed..

[3] Maruo S., Nakamura O., Kawata S. (1997). Three-dimensional microfabrication with two-photon-absorbed photopolymerization. Opt. Lett..

[4] Malinauskas M., Farsari M., Piskarskas A., Juodkazis S. (2013). Ultrafast laser nanostructuring of photopolymers: A decade of advances. Phys. Rep..

[5] Duan B., Wang M., Zhou W.Y., Cheung W.L., Li Z.Y., Lu W.W. (2010). Three-dimensional nanocomposite scaffolds fabricated via selective laser sintering for bone tissue engineering. Acta Biomater..

[6] Diermann S.H., Lu M., Zhao Y., Vandi L.J., Dargusch M., Huang H. (2018). Synthesis, microstructure, and mechanical behaviour of a unique porous PHBV scaffold manufactured using selective laser sintering. J. Mech. Behav. Biomed. Mater..

[7] Feng P., Kong Y., Yu L., Li Y., Gao C., Peng S., Pan H., Zhao Z., Shuai C. (2019). Molybdenum disulfide nanosheets embedded with nanodiamond particles: Co-dispersion nanostructures as reinforcements for polymer scaffolds. Appl. Mater. Today.

[8] Patel R., Monticone D., Lu M., Grøndahl L., Huang H. (2021). Hydrolytic degradation of porous poly(hydroxybutyrate-co-hydroxyvalerate) scaffolds manufactured using selective laser sintering. Polym. Degrad. Stab..

[9] Satoshi K., Hong-Bo S., Tomokazu T., Kenji T. (2001). Finer features for functional microdevices. Nature.

[10] Sun H.B., Kawata S. (2004). Two-photon photopolymerization and 3D lithographic microfabrication. Adv. Polym. Sci..

[11] Carlotti M., Mattoli V. (2019). Functional Materials for Two-Photon Polymerization in Microfabrication. Small.

[12] Ovsianikov A., Viertl J., Chichkov B., Oubaha M., MacCraith B., Sakellari I., Giakoumaki A., Gray D., Vamvakaki M., Farsari M. (2008). Ultra-low shrinkage hybrid photosensitive material for two-photon polymerization microfabrication. ACS Nano.

[13] Sakellari I., Kabouraki E., Gray D., Purlys V., Fotakis C., Pikulin A., Bityurin N., Vamvakaki M., Farsari M. (2012). Diffusion-assisted high-resolution direct femtosecond laser writing. ACS Nano.

[14] Cônsoli P.M., Otuka A.J.G., Balogh D.T., Mendonça C.R. (2018). Feature size reduction in two-photon polymerization by optimizing resin composition. J. Polym. Sci. Part B Polym. Phys..

[15] Seet K.K., Mizeikis V., Matsuo S., Juodkazis S., Misawa H. (2005). Three-dimensional spiral-architecture photonic crystals obtained by direct laser writing. Adv. Mater..

[16] Dietrich P.I., Blaicher M., Reuter I., Billah M., Hoose T., Hofmann A., Caer C., Dangel R., Offrein B., Troppenz U. (2018). In situ 3D nanoprinting of free-form coupling elements for hybrid photonic integration. Nat. Photonics.

[17] Tomazio N.B., Otuka A.J.G., Almeida G.F.B., Roselló-Mechó X., Andrés M.V., Mendonça C.R. (2017). Femtosecond laser fabrication of high-Q whispering gallery mode microresonators via two-photon polymerization. J. Polym. Sci. Part B Polym. Phys..

[18] Lindenmann N., Dottermusch S., Goedecke M.L., Hoose T., Billah M.R., Onanuga T.P., Hofmann A., Freude W., Koos C. (2015). Connecting silicon photonic circuits to multicore fibers by photonic wire bonding. J. Light. Technol..

[19] Jimenez Gordillo O.A., Chaitanya S., Chang Y.-C., Dave U.D., Mohanty A., Lipson M. (2019). Plug-and-play fiber to waveguide connector. Opt. Express.

[20] Pitts J.D., Campagnola P.J., Epling G.A., Goodman S.L. (2000). Submicron multiphoton free-form fabrication of proteins and polymers: Studies of reaction efficiencies and applications in sustained release. Macromolecules.

[21] Tayalia P., Mendonca C.R., Baldacchini T., Mooney D.J., Mazur E. (2008). 3D cell-migration studies using two-photon engineered polymer scaffolds. Adv. Mater..

[22] Gittard S.D., Ovsianikov A., Akar H., Chichkov B., Monteiro-Riviere N.A., Stafslien S., Chisholm B., Shin C.C., Shih C.M., Lin S.J. (2010). Two photon polymerization-micromolding of polyethylene glycol-gentamicin sulfate microneedles. Adv. Eng. Mater..

[23] Maruo S., Ikuta K., Korogi H. (2003). Submicron manipulation tools driven by light in a liquid. Appl. Phys. Lett..

[24] Maruo S., Ikuta K., Korogi H. (2003). Force-controllable, optically driven micromachines fabricated by single-step two-photon microstereolithography. J. Microelectromechanical Syst..

[25] Galajda P., Ormos P. (2001). Complex micromachines produced and driven by light. Appl. Phys. Lett..

[26] Zhang S., Tang S.J., Feng S., Xiao Y.F., Cui W., Wang X., Sun W., Ye J., Han P., Zhang X. (2019). High-Q Polymer Microcavities Integrated on a Multicore Fiber Facet for Vapor Sensing. Adv. Opt. Mater..

[27] Von Freymann G., Ledermann A., Thiel M., Staude I., Essig S., Busch K., Wegener M. (2010). Three-dimensional nanostructures for photonics. Adv. Funct. Mater..

[28] Klein S., Barsella A., Leblond H., Bulou H., Fort A., Andraud C., Lemercier G., Mulatier J.C., Dorkenoo K. (2005). One-step waveguide and optical circuit writing in photopolymerizable materials processed by two-photon absorption. Appl. Phys. Lett..

[29] Lindenmann N., Balthasar G., Hillerkuss D., Schmogrow R., Jordan M., Leuthold J., Freude W., Koos C. (2012). Photonic wire bonding: A novel concept for chip-scale interconnects. Opt. Express.

[30] Ams M., Marshall G.D., Dekker P., Piper J.A., Withford M.J. (2009). Ultrafast laser written active devices. Laser Photonics Rev..

[31] Tomazio N.B., De Boni L., Mendonca C.R. (2017). Low threshold Rhodamine-doped whispering gallery mode microlasers fabricated by direct laser writing. Sci. Rep..

[32] Malinauskas M., Rekštyte S., Lukoševičius L., Butkus S., Balčiunas E., Pečiukaityte M., Baltriukiene D., Bukelskiene V., Butkevičius A., Kucevičius P. (2014). 3D microporous scaffolds manufactured via combination of fused filament fabrication and direct laser writing ablation. Micromachines.

[33] Mačiulaitis J., Deveikyte M., Rekštyte S., Bratchikov M., Darinskas A., Šimbelyte A., Daunoras G., Laurinavičiene A., Laurinavičius A., Gudas R. (2015). Preclinical study of SZ2080 material 3D microstructured scaffolds for cartilage tissue engineering made by femtosecond direct laser writing lithography. Biofabrication.

[34] Maruo S., Fourkas J.T. (2008). Recent progress in multiphoton microfabrication. Laser Photonics Rev..

[35] Maruo S., Inoue H. (2006). Optically driven micropump produced by three-dimensional two-photon microfabrication. Appl. Phys. Lett..

[36] Boyd R.W. (2008). Nonlinear Optics.

[37] Pawlicki M., Collins H.A., Denning R.G., Anderson H.L. (2009). Two-photon absorption and the design of two-photon dyes. Angew. Chemie Int. Ed..

[38] Correa D.S., De Boni L., Otuka A.J.G., Tribuzi V., Mendonça C.R. (2012). Two-photon polymerization fabrication of doped microstructures. Polymerization.

[39] Karotki A., Drobizhev M., Kruk M., Spangler C., Nickel E., Mamardashvili N., Rebane A. (2003). Enhancement of two-photon absorption in tetrapyrrolic compounds. J. Opt. Soc. Am. B.

[40] Tomazio N.B. (2020). Direct Laser Writing of High-Q Polymeric Microresonators for Photonics. Ph.D. Thesis.

[41] Bhawalkar J.D., He G.S., Prasad P.N. (1996). Nonlinear multiphoton processes in organic and polymeric materials. Rep. Prog. Phys..

[42] Juodkazis S., Mizeikis V., Seet K.K., Miwa M., Misawa H. (2005). Two-photon lithography of nanorods in SU-8 photoresist. Nanotechnology.

[43] Sun H.B., Takada K., Kim M.S., Lee K.S., Kawata S. (2003). Scaling laws of voxels in two-photon photopolymerization nanofabrication. Appl. Phys. Lett..

[44] Schizas C., Melissinaki V., Gaidukeviciute A., Reinhardt C., Ohrt C., Dedoussis V., Chichkov B.N., Fotakis C., Farsari M., Karalekas D. (2010). On the design and fabrication by two-photon polymerization of a readily assembled micro-valve. Int. J. Adv. Manuf. Technol..

[45] Ovsianikov A., Gaidukeviciute A., Chichkov B.N., Oubaha M., MacCraith B.D., Sakellari I., Giakoumaki A., Gray D., Vamvakaki M., Farsari M. (2008). Two-photon polymerization of hybrid sol-gel materials for photonics applications. Laser Chem..

[46] Ovsianikov A., Mironov V., Stampf J., Liska R. (2012). Engineering 3D cell-culture matrices: Multiphoton processing technologies for biological and tissue engineering applications. Expert Rev. Med. Dev..

[47] Fowles G.R. (1969). Introduction to Modern Optics.

[48] Reinhardt C., Ovsianikov A., Passinger S., Chichkov B.N. (2007). Fabrication of micromechanical and microoptical systems by two-photon polymerization. Proc. SPIE 6466 MOEMS Miniaturized Syst. VI.

[49] Conradie E.H., Moore D.F. (2002). SU-8 thick photoresist processing as a functional material for MEMS applications. J. Micromech. Microeng..

[50] Baldacchini T., LaFratta C.N., Farrer R.A., Teich M.C., Saleh B.E.A., Naughton M.J., Fourkas J.T. (2004). Acrylic-based resin with favorable properties for three-dimensional two-photon polymerization. J. Appl. Phys..

[51] Li L., Gershgoren E., Kumi G., Chen W.Y., Ho P.T., Herman W.N., Fourkas J.T. (2008). High-performance microring resonators fabricated with multiphoton absorption polymerization. Adv. Mater..

[52] Li N., Driscoll M., Kumi G., Hernandez R., Gaskell K.J., Losert W., Fourkas J.T. (2008). Binary and gray-scale patterning of chemical functionality on polymer films. J. Am. Chem. Soc..

[53] Otuka A.J.G., Tribuzi V., Corrêa D.S., Mendonça C.R. (2012). Emission features of microstructures fabricated by two-photon polymerization containing three organic dyes. Opt. Mater. Express.

[54] Fonseca R.D., Correa D.S., Paris E.C., Tribuzi V., Dev A., Voss T., Aoki P.H.B., Constantino C.J.L., Mendonca C.R. (2014). Fabrication of zinc oxide nanowires/polymer composites by two-photon polymerization. J. Polym. Sci. Part B Polym. Phys..

[55] Henrique F.R., Mendonca C.R. (2016). Local excitation and collection in polymeric fluorescent microstructures. Opt. Mater..

[56] Otuka A.J.G., Corrêa D.S., Fontana C.R., Mendonça C.R. (2014). Direct laser writing by two-photon polymerization as a tool for developing microenvironments for evaluation of bacterial growth. Mater. Sci. Eng. C.

[57] Teh W.H., Dürig U., Drechsler U., Smith C.G., Güntherodt H.J. (2005). Effect of low numerical-aperture femtosecond two-photon absorption on (SU-8) resist for ultrahigh-aspect-ratio microstereolithography. J. Appl. Phys..

[58] Serbin J., Egbert A., Ostendorf A., Chichkov B.N., Houbertz R., Domann G., Schulz J., Cronauer C., Fröhlich L., Popall M. (2003). Femtosecond laser-induced two-photon polymerization of inorganic–organic hybrid materials for applications in photonics. Opt. Lett..

[59] Harnisch E., Russew M., Klein J., König N., Crailsheim H., Schmitt R. (2015). Optimization of hybrid polymer materials for 2PP and fabrication of individually designed hybrid microoptical elements thereof. Opt. Mater. Express.

[60] Mir S.H., Nagahara L.A., Thundat T., Mokarian-Tabari P., Furukawa H., Khosla A. (2018). Review—Organic-Inorganic Hybrid Functional Materials: An Integrated Platform for Applied Technologies. J. Electrochem. Soc..

[61] Jiang L.J., Zhou Y.S., Xiong W., Gao Y., Huang X., Jiang L., Baldacchini T., Silvain J.-F., Lu Y.F. (2014). Two-photon polymerization: Investigation of chemical and mechanical properties of resins using Raman microspectroscopy. Opt. Lett..

[62] He Z., Lee Y.-H., Chanda D., Wu S.-T. (2018). Adaptive liquid crystal microlens array enabled by two-photon polymerization. Opt. Express.

[63] Wu Z.L., Qi Y.N., Yin X.J., Yang X., Chen C.M., Yu J.Y., Yu J.C., Lin Y.M., Hui F., Liu P.L. (2019). Polymer-Based Device Fabrication and Applications Using Direct Laser Writing Technology. Polymers.

[64] Doraiswamy A., Ovsianikov A., Gittard S.D., Monteiro-Riviere N.A., Crombez R., Montalvo E., Shen W., Chichkov B.N., Narayan R.J. (2010). Fabrication of microneedles using two photon polymerization for transdermal delivery of nanomaterials. J. Nanosci. Nanotechnol..

[65] Huang K.M., Tsai S.C., Lee Y.K., Yuan C.K., Chang Y.C., Chiu H.L., Chung T.T., Liao Y.C. (2017). Selective metallic coating of 3D-printed microstructures on flexible substrates. RSC Adv..

[66] Ovsianikov A., Deiwick A., Van Vlierberghe S., Dubruel P., Möller L., Drager G., Chichkov B. (2011). Laser fabrication of three-dimensional CAD scaffolds from photosensitive gelatin for applications in tissue engineering. Biomacromolecules.

[67] Billiet T., Vandenhaute M., Schelfhout J., Van Vlierberghe S., Dubruel P. (2012). A review of trends and limitations in hydrogel-rapid prototyping for tissue engineering. Biomaterials.

[68] Claeyssens F., Hasan E., Gaidukeviciute A., Achilleos D., Ranella A., Reinhardt C., Ovsianikov A., Shizhou X., Fotakis C., Vamvakaki M. (2009). Three-Dimensional Biodegradable Structures Fabricated by Two-Photon Polymerization. Langmuir.

[69] Yao H., Wang J., Mi S. (2017). Photo processing for biomedical hydrogels design and functionality: A review. Polymers.

[70] Dobos A., Gantner F., Markovic M., van Hoorick J., Tytgat L., van Vlierberghe S., Ovsianikov A. (2020). On-chip high-definition bioprinting of microvascular structures. Biofabrication.

[71] Weisgrab G., Guillaume O., Guo Z., Heimel P., Slezak P., Poot A., Grijpma D., Ovsianikov A. (2020). 3D printing of large-scale and highly porous biodegradable tissue engineering scaffolds from poly(trimethylene-carbonate) using two-photon-polymerization. Biofabrication.

[72] Whitby R., Ben-Tal Y., MacMillan R., Janssens S., Raymond S., Clarke D., Jin J., Kay A., Simpson M.C. (2017). Photoinitiators for two-photon polymerisation: Effect of branching and viscosity on polymerisation thresholds. RSC Adv..

[73] Huang Z., Deng Y. (2020). Two-photon polymerization nanolithography technology for fabrication of stimulus-responsive micro/nano-structures for biomedical applications 2 TPP PIs for biomedical applications. Nanotechnol. Rev..

[74] Flory P. (1953). Principles of Polymer Chemistry.

[75] Mendonca C.R., Correa D.S., Baldacchini T., Tayalia P., Mazur E. (2008). Two-photon absorption spectrum of the photoinitiator Lucirin TPO-L. Appl. Phys. A Mater. Sci. Process..

[76] Temel G., Enginol B., Aydin M., Balta D.K., Arsu N. (2011). Photopolymerization and photophysical properties of amine linked benzophenone photoinitiator for free radical polymerization. J. Photochem. Photobiol. A Chem..

[77] Fouassier J.-P. (1995). Photoinitiation, Photopolymerization, and Photocuring: Fundamentals and Applications.

[78] Michaudel Q., Kottisch V., Fors B.P. (2017). Cationic Polymerization: From Photoinitiation to Photocontrol. Angew. Chemie Int. Ed..

[79] Žukauskas A., Malinauskas M., Kontenis L., Purlys V., Paipulas D., Vengris M., Gadonas R. (2010). Organic dye doped microstructures for optically active functional devices fabricated via two-photon polymerization technique. Lith. J. Phys..

[80] Otuka A.J.G., Torres B.B.M., Dipold J., Balogh D.T., Tribuzi V., De Boni L., Mendonça C.R. (2020). Three-dimensional structures fabricated after laser-induced free radical generation in azoaromatic compounds. Opt. Mater. Express.

[81] Ramasubramaniam R., Chen J., Liu H. (2003). Homogeneous carbon nanotube/polymer composites for electrical applications. Appl. Phys. Lett..

[82] Hone J., Llaguno M.C., Biercuk M.J., Johnson A.T., Batlogg B., Benes Z., Fischer J.E. (2002). Thermal properties of carbon nanotubes and nanotube-based materials. Appl. Phys. A Mater. Sci. Process..

[83] Peng X., Komatsu N., Bhattacharya S., Shimawaki T., Aonuma S., Kimura T., Osuka A. (2007). Optically active single-walled carbon nanotubes. Nat. Nanotechnol..

[84] Ci L., Suhr J., Pushparaj V., Zhang X., Ajayan P.M. (2008). Continuous Carbon Nanotube Reinforced Composites Continuous Carbon Nanotube. Nano Lett..

[85] Otuka A.J.G., Tribuzi V., Cardoso M.R., De Almeida G.F.B., Zanatta A.R., Corrêa D.S., Mendonça C.R. (2015). Single-walled Carbon nanotubes functionalized with carboxylic acid for fabricating polymeric composite microstructures. J. Nanosci. Nanotechnol..

[86] Guo Q., Xiao S., Aumann A., Jaeger M., Chakif M., Ghadiri R., Esen C., Ma M., Ostendorf A. (2012). Using laser microfabrication to write conductive polymer/swnts nanocomposites. J. Laser Micro Nanoeng..

[87] Ushiba S., Shoji S., Masui K., Kuray P., Kono J., Kawata S. (2013). 3D microfabrication of single-wall carbon nanotube/polymer composites by two-photon polymerization lithography. Carbon N. Y..

[88] Ushiba S., Shoji S., Masui K., Kono J., Kawata S. (2014). Direct laser writing of 3D architectures of aligned carbon nanotubes. Adv. Mater..

[89] Xiong W., Liu Y., Jiang L.J., Zhou Y.S., Li D.W., Jiang L., Silvain J.F., Lu Y.F. (2016). Laser-Directed Assembly of Aligned Carbon Nanotubes in Three Dimensions for Multifunctional Device Fabrication. Adv. Mater..

[90] Formanek F., Takeyasu N., Tanaka T., Chiyoda K., Ishikawa A., Kawata S. (2006). Three-dimensional fabrication of metallic nanostructures over large areas by two-photon polymerization. Opt. Express.

[91] Ovsianikov A., Gruene M., Pflaum M., Koch L., Maiorana F., Wilhelmi M., Haverich A., Chichkov B. (2010). Laser printing of cells into 3D scaffolds. Biofabrication.

[92] Paula K.T., Tomazio N.B., Salas O.I.A., Otuka A.J.G., Almeida J.M.P., Andrade M.B., Vieira N.C.S., Balogh D.T., Mendonça C.R. (2021). Femtosecond-laser selective printing of graphene oxide and PPV on polymeric microstructures. J. Mater. Sci..

[93] Xia H., Wang J., Tian Y., Chen Q.D., Du X.B., Zhang Y.L., He Y., Sun H.B. (2010). Ferrofluids for fabrication of remotely controllable micro-nanomachines by two-photon polymerization. Adv. Mater..

[94] Chung T.-T., Tseng C.-L., Hung C.-P., Lin C.-L., Baldeck P.L. (2013). Design and Two-Photon Polymerization of Complex Functional Micro-Objects for Lab-on-a-Chip: Rotating Micro-Valves. J. Neurosci. Neuroeng..

[95] Righini G.C., Dumeige Y., Féron P., Ferrari M., Nunzi Conti G., Ristic D., Soria S. (2011). Whispering gallery mode microresonators: Fundamentals and applications. Riv. del Nuovo Cim..

[96] Vahala K.J. (2003). Optical microcavities. Nature.

[97] Kippenberg T.J., Holzwarth R., Diddams S.A. (2011). Microresonator-based optical frequency combs. Science.

[98] Ward J., Benson O. (2011). WGM microresonators: Sensing, lasing and fundamental optics with microspheres. Laser Photonics Rev..

[99] He L., Özdemir Ş.K., Yang L. (2013). Whispering gallery microcavity lasers. Laser Photonics Rev..

[100] Foreman M.R., Swaim J.D., Vollmer F. (2015). Whispering gallery mode sensors. Adv. Opt. Photonics.

[101] Zhu J., Ozdemir S.K., Xiao Y.F., Li L., He L., Chen D.R., Yang L. (2010). On-chip single nanoparticle detection and sizing by mode splitting in an ultrahigh-Q microresonator. Nat. Photonics.

[102] Baaske M.D., Foreman M.R., Vollmer F. (2014). Single-molecule nucleic acid interactions monitored on a label-free microcavity biosensor platform. Nat. Nanotechnol..

[103] Heylman K.D., Thakkar N., Horak E.H., Quillin S.C., Cherqui C., Knapper K.A., Masiello D.J., Goldsmith R.H. (2016). Optical microresonators as single-particle absorption spectrometers. Nat. Photonics.

[104] Del’Haye P., Schliesser A., Arcizet O., Wilken T., Holzwarth R., Kippenberg T.J. (2007). Optical frequency comb generation from a monolithic microresonator. Nature.

[105] Jiang X.F., Xiao Y.F., Zou C.L., He L., Dong C.H., Li B.B., Li Y., Sun F.W., Yang L., Gong Q. (2012). Highly unidirectional emission and ultralow-threshold lasing from on-chip ultrahigh-Q microcavities. Adv. Mater..

[106] Peng B., Özdemir S.K., Lei F., Monifi F., Gianfreda M., Long G.L., Fan S., Nori F., Bender C.M., Yang L. (2014). Parity-time-symmetric whispering-gallery microcavities. Nat. Phys..

[107] Spillane S.M., Kippenberg T.J., Vahala K.J. (2002). Ultralow-threshold Raman laser using a spherical dielectric microcavity. Nature.

[108] Ma Z., Chen J.-Y., Li Z., Tang C., Sua Y.M., Fan H., Huang Y.-P. (2020). Ultrabright Quantum Photon Sources on Chip. Phys. Rev. Lett..

[109] Matsko A.B., Ilchenko V.S. (2006). Optical resonators with whispering-gallery modes—Part I: Basics. IEEE J. Sel. Top. Quantum Electron..

[110] Roselló-Mechó X. (2019). Whispering Gallery Modes: Advanced Photonic Applications.

[111] Zamora V., Díez A., Andrés M.V., Gimeno B. (2011). Cylindrical optical microcavities: Basic properties and sensor applications. Proceedings of the Photonics and Nanostructures—Fundamentals and Applications.

[112] Saleh B.E.A., Teich M.C. (1991). Fundamentals of Photonics.

[113] Pollock C.R., Lipson M. (2003). Integrated Photonics.

[114] Gorodetsky M.L., Savchenkov A.A., Ilchenko V.S. (1996). Ultimate Q of optical microsphere resonators. Opt. Lett..

[115] Chen D., Kovach A., Shen X., Poust S., Armani A.M. (2017). On-Chip Ultra-High-Q Silicon Oxynitride Optical Resonators. ACS Photonics.

[116] Vernooy D.W., Ilchenko V.S., Mabuchi H., Streed E.W., Kimble H.J. (1998). High-Q measurements of fused-silica microspheres in the near infrared. Opt. Lett..

[117] Rukhlenko I.D., Premaratne M., Agrawal G.P. (2010). Analytical study of optical bistability in silicon ring resonators. Opt. Lett..

[118] Rabiei P., Steier W.H., Zhang C., Dalton L.R. (2002). Polymer micro-ring filters and modulators. J. Light. Technol..

[119] Grossmann T., Hauser M., Beck T., Gohn-Kreuz C., Karl M., Kalt H., Vannahme C., Mappes T. (2010). High-Q conical polymeric microcavities. Appl. Phys. Lett..

[120] Chao C.Y., Guo L.J. (2002). Polymer microring resonators fabricated by nanoimprint technique. J. Vac. Sci. Technol. B Microelectron. Nanom. Struct..

[121] Martin A.L., Armani D.K., Yang L., Vahala K.J. (2004). Replica-molded high-Q polymer microresonators. Opt. Lett..

[122] Dong C.H., He L., Xiao Y.F., Gaddam V.R., Ozdemir S.K., Han Z.F., Guo G.C., Yang L. (2009). Fabrication of high- Q polydimethylsiloxane optical microspheres for thermal sensing. Appl. Phys. Lett..

[123] Zhang C., Zou C.L., Zhao Y., Dong C.H., Wei C., Wang H., Liu Y., Guo G.C., Yao J., Zhao Y.S. (2015). Organic printed photonics: From microring lasers to integrated circuits. Sci. Adv..

[124] Saetchnikov A.V., Tcherniavskaia E.A., Saetchnikov V.A., Ostendorf A. (2020). A Laser Written 4D Optical Microcavity for Advanced Biochemical Sensing in Aqueous Environment. J. Light. Technol..

[125] Ku J.-F., Chen Q.-D., Zhang R., Sun H.-B. (2011). Whispering-gallery-mode microdisk lasers produced by femtosecond laser direct writing. Opt. Lett..

[126] Schumann M., Bückmann T., Gruhler N., Wegener M., Pernice W. (2014). Hybrid 2D-3D optical devices for integrated optics by direct laser writing. Light Sci. Appl..

[127] Sun Y.L., Hou Z.S., Sun S.M., Zheng B.Y., Ku J.F., Dong W.F., Chen Q.D., Sun H.B. (2015). Protein-Based Three-Dimensional Whispering-Gallery-Mode Micro-Lasers with Stimulus-Responsiveness. Sci. Rep..

[128] Wei H., Krishnaswamy S. (2016). Direct Laser Writing Polymer Micro-Resonators for Refractive Index Sensors. IEEE Photonics Technol. Lett..

[129] Wei H., Krishnaswamy S. (2017). Polymer micro-ring resonator integrated with a fiber ring laser for ultrasound detection. Opt. Lett..

[130] Hou Z.S., Huang Q.L., Zhan X.P., Li A.W., Xu H.L. (2017). Real 3D microsphere lasers by femtosecond laser processing. RSC Adv..

[131] Nocentini S., Riboli F., Burresi M., Martella D., Parmeggiani C., Wiersma D.S. (2018). Three-Dimensional Photonic Circuits in Rigid and Soft Polymers Tunable by Light. ACS Photonics.

[132] Kelemen L., Lepera E., Horváth B., Ormos P., Osellame R., Martínez Vázquez R. (2019). Direct writing of optical microresonators in a lab-on-a-chip for label-free biosensing. Lab Chip.

[133] Siegle T., Schierle S., Kraemmer S., Richter B., Wondimu S.F., Schuch P., Koos C., Kalt H. (2017). Photonic molecules with a tunable inter-cavity gap. Light Sci. Appl..

[134] Siegle T., Remmel M., Krämmer S., Kalt H. (2017). Split-disk micro-lasers: Tunable whispering gallery mode cavities. APL Photonics.

[135] Schell A.W., Kaschke J., Fischer J., Henze R., Wolters J., Wegener M., Benson O. (2013). Three-dimensional quantum photonic elements based on single nitrogen vacancy-centres in laser-written microstructures. Sci. Rep..

[136] Flatae A.M., Burresi M., Zeng H., Nocentini S., Wiegele S., Parmeggiani C., Kalt H., Wiersma D. (2015). Optically controlled elastic microcavities. Light Sci. Appl..

[137] Tomazio N.B., Paula K.T., Henrique F.R., Andrade M.B., Roselló-Mechó X., Delgado-Pinar M., Andrés M.V., Mendonca C.R. (2020). Mode cleaning in graphene oxide-doped polymeric whispering gallery mode microresonators. J. Mater. Chem. C.

[138] Sherwood T., Young A.C., Takayesu J., Jen A.K.Y., Dalton L.R., Chen A. (2005). Microring resonators on side-polished optical fiber. IEEE Photonics Technol. Lett..

[139] Liu Z.P., Li Y., Xiao Y.F., Li B.B., Jiang X.F., Qin Y., Feng X.B., Yang H., Gong Q. (2010). Direct laser writing of whispering gallery microcavities by two-photon polymerization. Appl. Phys. Lett..

[140] Grossmann T., Schleede S., Hauser M., Beck T., Thiel M., von Freymann G., Mappes T., Kalt H. (2011). Direct laser writing for active and passive high-Q polymer microdisks on silicon. Opt. Express.

[141] Li Z., Liao C., Wang J., Li Z., Zhou P., Wang Y., Wang Y. (2019). Femtosecond Laser Microprinting of a Fiber Whispering Gallery Mode Resonator for Highly-Sensitive Temperature Measurements. J. Light. Technol..

[142] Zhan X.P., Xu Y.X., Xu H.L., Huang Q.L., Hou Z.S., Fang W., Chen Q.D., Sun H.B. (2017). Toward On-Chip Unidirectional and Single-Mode Polymer Microlaser. J. Light. Technol..

[143] Schäfer F.P. (1990). Dye Lasers.

[144] Casey K.G., Quitevis E.L. (1988). Effect of solvent polarity on nonradiative processes in xanthene dyes: Rhodamine B in normal alcohols. J. Phys. Chem..

[145] Grossmann T., Klinkhammer S., Hauser M., Floess D., Beck T., Vannahme C., Mappes T., Lemmer U., Kalt H. (2011). Strongly confined, low-threshold laser modes in organic semiconductor microgoblets. Opt. Express.

[146] Grossmann T., Schleede S., Hauser M., Christiansen M.B., Vannahme C., Eschenbaum C., Klinkhammer S., Beck T., Fuchs J., Nienhaus G.U. (2010). Low-threshold conical microcavity dye lasers. Appl. Phys. Lett..

[147] Zhan X.P., Ku J.F., Xu Y.X., Zhang X.L., Zhao J., Fang W., Xu H.L., Sun H.B. (2015). Unidirectional lasing from a spiral-shaped microcavity of dye-doped polymers. IEEE Photonics Technol. Lett..

[148] Ku J., Chen Q., Ma X., Yang Y., Huang Y., Member S., Xu H., Sun H. (2015). Photonic-Molecule Single-Mode Laser. IEEE Photonics Technol. Lett..

[149] Zamora V., Díez A., Andrés M.V., Gimeno B. (2007). Refractometric sensor based on whispering-gallery modes of thin capillarie. Opt. Express.

[150] Avila O.I., Otuka A.J.G., Tribuzi V., Freitas L.M., Serafim R.B., Moraes M.H., Espreafico E.M., Valente V., Fontana C.R., Mendonça C.R. (2014). Fabrication of Microenvironments with Different Geometrical Features for Cell Growth Studies. J. Laser Micro Nanoeng..

[151] Bakhtina N.A., Müller M., Wischnewski H., Arora R., Ciaudo C. (2021). 3D Synthetic Microstructures Fabricated by Two-Photon Polymerization Promote Homogeneous Expression of NANOG and ESRRB in Mouse Embryonic Stem Cells. Adv. Mater. Interfaces.

[152] Sabaté Rovira D., Nielsen H.M., Taboryski R., Bunea A.I. (2021). Additive manufacturing of polymeric scaffolds for biomimetic cell membrane engineering. Mater. Des..

[153] Koroleva A., Deiwick A., El-Tamer A., Koch L., Shi Y., Estévez-Priego E., Ludl A.A., Soriano J., Guseva D., Ponimaskin E. (2021). In vitro development of human iPSC-derived functional neuronal networks on laser-fabricated 3D scaffolds. ACS Appl. Mater. Interfaces.

[154] Gallegos A.M.A., Carrera S.H., Parra R., Keshavarz T., Iqbal H.M.N. (2016). Bacterial cellulose: A sustainable source to develop value-added products—A review. BioResources.

[155] Wang J., Tavakoli J., Tang Y. (2019). Bacterial cellulose production, properties and applications with different culture methods—A review. Carbohydr. Polym..

[156] Otuka A.J.G., Domeneguetti R.R., Moraes J.Q.R., Balogh D.T., Ribeiro S.J.L., Mendonça C.R. (2021). Bacterial cellulose growth on 3D acrylate-based microstructures fabricated by two-photon polymerization. J. Phys. Photonics.

[157] Sun X., Hourwitz M.J., Baker E.M., Schmidt B.U.S., Losert W., Fourkas J.T. (2018). Replication of biocompatible, nanotopographic surfaces. Sci. Rep..

